# CircMAN1A2 Levels Determine GBM Susceptibility to TMZ in a Pathway Involving TEP1‐ and KEAP1‐Mediated NRF2 Degradation Leading to Ferroptosis

**DOI:** 10.1111/cns.70489

**Published:** 2025-06-30

**Authors:** Xinqiao Li, Jinpeng Hu, Wei Zheng, Zongming Fan, Hao Chi, Hao Li, Yongfeng Wang, Zhitao Jing

**Affiliations:** ^1^ Department of Neurosurgery The First Hospital of China Medical University Heping District, Shenyang China; ^2^ Department of Histology and Embryology Basic Medical College, China Medical University Shenyang China; ^3^ Department of Cardiac Surgery Henan Provincial Chest Hospital, Chest Hospital of Zhengzhou University Zhengzhou China; ^4^ Department of Anesthesiology Henan Provincial Chest Hospital, Chest Hospital of Zhengzhou University Zhengzhou China; ^5^ School of Clinical Medical, Southwest Medical University Luzhou China; ^6^ Department of Urology, Urologic Surgery Center Xinqiao Hospital, Third Military Medical University (Army Medical University) Chongqing China; ^7^ Department of Radiology The First Hospital of China Medical University Shenyang China

**Keywords:** ANXA1, circRNA, glioma stem cells, KEAP1‐NRF2 pathway, tumor‐associated macrophages

## Abstract

**Background:**

Glioblastoma (GBM) is a highly aggressive brain tumor with poor prognosis and resistance to temozolomide (TMZ). The role of downregulated circular RNAs (circRNAs) in GBM progression remains unclear.

**Methods:**

CircRNA sequencing and public dataset analysis identified dysregulated circRNAs in GBM. Functional assays were conducted in patient‐derived glioma stem‐like cells (GSCs) with circMAN1A2 overexpression or knockdown. Ferroptosis‐related experiments, protein interaction assays, and in vivo mouse models were used to explore mechanisms and therapeutic effects.

**Results:**

CircMAN1A2 was significantly downregulated in GBM tissues and inversely correlated with WHO grade and patient survival. Overexpression of circMAN1A2 inhibited GSC proliferation, stemness, invasion, and reversed TMZ resistance by inducing ferroptosis. Mechanistically, circMAN1A2 was regulated by PRPF40B and directly bound to TEP1, disrupting its interaction with KEAP1, thereby promoting NRF2 degradation and ferroptosis. NRF2 upregulated ANXA1, which promoted tumor‐associated macrophage (TAM) recruitment and M2 polarization. In vivo, circMAN1A2 overexpression suppressed tumor growth, enhanced TMZ sensitivity, and reduced NRF2/ANXA1 expression, effects reversed by TEP1.

**Conclusions:**

CircMAN1A2 suppresses GBM progression and TMZ resistance by inducing ferroptosis and modulating the TEP1–KEAP1–NRF2–ANXA1 axis. It represents a potential therapeutic target in GBM.

## Graphical abstract

PRPF40B promotes circMAN1A2 expression, inhibiting TEP1‐KEAP1 complex formation, leading to NRF2 degradation and ferroptosis in GSCs. Without circMAN1A2, NRF2 activation inhibits ferroptosis and promotes TAMs' M2 polarization.

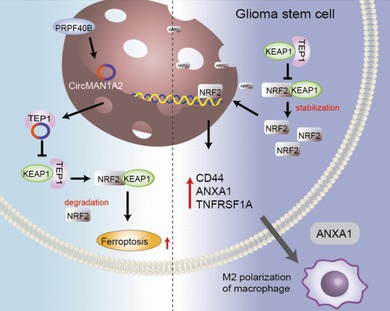

**Abbreviations**
ANXA1: Annexin A1
CGGA: Chinese Glioma Genome Atlas
ChIP: Chromatin Immunoprecipitation
CircRNA: circular RNA
COIP: co‐immunoprecipitation
DMEM: Dulbecco's Modified Eagle Medium
EdU: 5‐ethynyl‐2‐deoxyuridine
ELISA: enzyme‐linked immunosorbent assay
FISH: Fluorescence in situ hybridization
GSH: glutathione
IHC: immunohistochemistry
KEAP1: Kelch Like ECH Associated Protein 1
MDA: malonaldehyde
miRNA: microRNA
NRF2: nuclear factor erythroid 2‐related factor 2
qRT‐PCR: real‐time quantitative reverse transcription PCR
RIP: RNA‐Binding Protein Immunoprecipitation
scRNA seq: single‐cell RNA sequencing
TAM: tumor‐associated macrophages
TCGA: The Cancer Genome Atlas
TEP1: Telomerase Associated Protein 1
TMZ: temozolomide
TNFRSF1A: TNF Receptor Superfamily Member 1A

## 1 Introduction

Glioblastoma, the most malignant and aggressive form of glioma [1], has the worst prognosis among these central nervous system tumors [2]. Even after comprehensive treatments like surgery, radiation therapy, and chemotherapy, the 5‐year survival rate of GBM has improved by only 3% [3]. Temozolomide is widely used as a first‐line chemotherapy drug for the treatment of GBM; however, nearly half of these patients develop resistance, limiting its efficacy [4]. A major contributing factor to this treatment failure is the high intratumoral and intertumoral heterogeneity of GBM [5]. This tumor type contains a subpopulation of glioma stem cells (GSCs) with stem cell‐like characteristics that contribute to tumor heterogeneity and display preferential resistance to radiotherapy and chemotherapy [6, 7, 8, 9]. Therefore, understanding the molecular mechanisms that maintain the stemness and promote the malignant progression of GSCs is crucial for identifying novel molecular targets and improving the prognosis of glioma patients.

Circular RNAs (circRNAs) are a class of endogenous non‐coding RNAs characterized by their covalently closed‐loop structure without 5′ caps or 3′ poly(A) tails [10]. This unique structure confers them high stability and resistance to exonucleases. circRNAs exhibit greater stability compared to linear RNAs, allowing them to exert various biological functions over extended periods [11]. Initially regarded as splicing artifacts, circRNAs have now emerged as important regulatory molecules in various physiological and pathological processes, including cancer. Recent studies have shown that circRNAs can function as microRNA sponges, interact with RNA‐binding proteins, and modulate transcription or even translation. In glioblastoma, certain circRNAs have been implicated in regulating stemness, therapeutic resistance, and immune modulation. However, the specific roles of many circRNAs, including their upstream regulators and downstream targets, remain poorly understood. In gliomas, circRNAs contribute to the progression of malignant biology through a multitude of mechanisms. In addition to the well‐established ceRNA mechanism, circRNAs can also function by binding to and stabilizing proteins, altering their cellular localization, and even translating into peptides and proteins [10, 12]. Currently, many circRNAs have been found to play various roles in the progression of gliomas. Here are a few examples: It has been reported that the RNA‐binding protein EIF4A3‐mediated circASAP1 is significantly upregulated in recurrent GBM and TMZ‐resistant cell lines. Importantly, it acts as a sponge for miR‐502‐5p, enhancing NRAS expression, activating the MEK1/ERK1 signaling pathway, and promoting TMZ resistance in GSCs [13]. U2AF65‐induced circNCAPG stabilizes the transcription factor RREB1 protein and promotes its nuclear translocation. This forms a positive feedback loop that activates the TGF‐β signaling and NESTIN expression, contributing to the malignant progression of GSCs [14]. In addition, high expression of circEHZ2 in GSCs translates into the EZH2‐92aa polypeptide, which stabilizes EZH2 protein and suppresses NKG2DLs expression, thereby inhibiting NK cell activation and facilitating immune escape in GSCs [15]. Therefore, a deeper understanding of the role and mechanisms of circRNAs in GSCs will not only contribute to our understanding of glioma development and progression but also provide more effective treatment options for glioma patients.

Telomerase Associated Protein 1 (TEP1) is a telomerase‐associated protein in mammals. Initially identified as a homolog of the p80 protein, TEP1 is involved in telomerase complex composition and acts as an RNA‐binding protein that interacts specifically with the telomerase RNA, which is critical for telomere maintenance and genome stability [16, 17]. Most tumor cells exhibit high levels of telomerase activity, which plays an important role in maintaining sustained tumor proliferation [18]. As an important component of the telomerase complex, TEP1 has emerged as a potential target for cancer therapy. Telomerase activation and p53 activation are involved in the transition from cellular immortalization to tumorigenicity. While p53 does not act directly on telomerase or its substrate DNA, it inhibits telomerase activity by preventing the formation of the telomerase complex through interaction with TEP1 [19]. miRNA‐380‐5p inhibits the expression of TEP1 by directly binding to its open reading frame, which in turn inhibits telomerase activity and induces apoptosis in diffuse malignant peritoneal mesothelioma cells [20]. Various studies have demonstrated the involvement of TEP1 in malignant tumor progression [21, 22, 23]. However, the role of TEP1 in the biological progression of gliomas remains unexplored. Exploring the molecular function of TEP1 in gliomas, especially in GSCs, could provide novel avenues for glioma therapy.

Macrophages are key components of the tumor immune microenvironment and display remarkable plasticity. Depending on the stimuli they encounter, macrophages can adopt different functional phenotypes, broadly classified into classically activated M1 macrophages with pro‐inflammatory and anti‐tumoral properties, and alternatively activated M2 macrophages, which are associated with tissue remodeling, immunosuppression, and tumor progression. In glioblastoma, tumor‐associated macrophages (TAMs), which are often skewed toward an M2‐like phenotype, contribute to an immunosuppressive microenvironment that supports tumor growth and resistance to therapy. Understanding the molecular cues that drive M2 polarization is essential for developing strategies to modulate TAMs for therapeutic benefit.

In this study, we identified a novel circRNA, hsa_circ_0000118, derived from the *MAN1A2* gene. This circRNA, downregulated in GBM, suppresses tumor growth and aggressiveness. We demonstrate that circMAN1A2 promotes the binding of KEAP1‐NRF2 by competitively binding to TEP1, resulting in NRF2 degradation. This, in turn, induces ferroptosis in glioma stem cells, suppressing their malignant phenotypes and reversing TMZ resistance. In addition, NRF2 transcriptional upregulation of ANXA1 expression promotes tumor‐associated macrophage recruitment and M2 polarization. Overall, our data suggest that circMAN1A2 functions as a tumor suppressor during GBM progression and represents a promising therapeutic target for targeting GSCs.

## 2 Methods

### 2.1 Clinical Samples and Data Sources

Glioma samples for circRNA sequencing (3 cases of paraneoplastic tissues and 3 cases of IDH wild‐type grade IV glioma tissues) were collected from the First Hospital of China Medical University. Glioma samples for circMAN1A2 expression were provided by the First Affiliated Hospital of China Medical University, including 10 cases of paraneoplastic normal tissue, 20 cases of WHO grade II, 25 cases of grade III, and 25 cases of grade IV. Our samples were collected between 2012 and 2020, and all samples were confirmed by two pathologists according to the 2016 WHO grading. Patient informed consent was obtained, and the study was approved by the Ethics Committee of the First Affiliated Hospital of China Medical University. Patient data is reflected in Table S1. The dataset GSE92322 containing 5 pairs of clinical samples was downloaded from the public database GEO for finding differentially expressed circRNAs in gliomas. For downstream raw data filtered by FastQC for quality control. Using Bowtie2 DNA comparison software to extract the unmatched sequences. Bowtie2 DNA comparison software was used to extract the unmatched sequences, and the circRNAs that existed in both algorithms were included in the study. Log2 transformation of circRNA BSJ reads into TPM values based on circRNA annotation information from circBase. Differentially expressed circRNAs were identified using the R package Limma, and |Log2FC| > 1 and *p* < 0.05 were considered differentially expressed circRNAs.

### 2.2 Data Sources

CircRNAseq dataset GSE92322, GBM single cell dataset GSE131928 downloaded from GEO (Gene Expression Omnibus) database. Public databases of glioma patient expression profiling data and survival information were obtained from CGGA (Chinese Glioma Genome Altas), TCGA (The Cancer Genome Atlas), Rembrandt dataset, and queried through the portal Gliovis. GEPIA2 database was used to query for gene correlations.

### 2.3 Bioinformatics Analysis

All analyses were performed based on R 4.1.3; the Limma package was used to identify differentially expressed circRNAs, the Seurat V4 pipeline was used for single‐cell analysis, ARACNe was used to construct gene regulatory networks, VIPER was used for protein activity analysis, and protein docking was used to analyze the strength of protein interactions.

### 2.4 Culture of Cells

Glioma stem cells (WHO IV: GSCL1, GSCL3, GSCL4, GSCL7, GSCL8, GSCL9) were isolated from fresh clinical GBM tissues. Extraction of patient‐derived primary glioma cells was performed as previously described [24]. The tissues were washed and mechanically minced. The tissue was then enzymatically digested into single cells using 0.25% trypsin (Gibco). Single cells were filtered through a 70 μm cell strainer and centrifuged (400 *g*) for 5 min. The cells were treated with erythrocyte lysate (Solarbio, Beijing, China) and centrifuged again. Finally, the obtained cells were cultured in serum‐free medium DMEM/F12 (Gibco) with the addition of B27 (2%, Gibco), recombinant human basic fibroblast growth factor (rh‐bFGF, 20 ng/mL, Gibco) and epidermal growth factor (rhEGF, 20 ng/mL, Gibco). The cells were incubated at 37°C, 5% CO_2_. Human monocyte cell line THP‐1 was purchased from the Chinese Academy of Sciences cell bank (Shanghai, China), which was primed with 5 nM PMA (Sigma) for 48 h to become monocyte‐derived macrophages. The THP‐1 cell was cultured in RPMI‐1640 medium (Gibco), containing 10% FBS and 1% penicillin/streptomycin. All cells were cultured at 37°C with 5% CO_2_. All cell lines and GSC studied were cultured for less than 20 generations.

### 2.5 RNase R Treatment

Total RNA was incubated with or without RNase R (3 U/mg, Epicenter Technologies, Madison, USA) for 30 min at 37°C. RNase R was used to confirm the existence of circMAN1A2 and eliminate the impact of linear MAN1A2.

### 2.6 Genomic DNA (gDNA) Extraction

Glioma stem cell spheroids were collected, washed three times in pre‐cooled PBS, and 30–50 spheroids were resuspended in 200 μL PBS. To the resuspension, add 4 μL of 100 mg/mL RNase A, vortex mixing, and leave at room temperature for 2 min to clear the RNA. The rest of the steps method was as described in the kit instructions (Beyotime).

### 2.7 Quantitative Real‐Time PCR

Total RNA was extracted with TRIzol (TaKaRa, Japan) following manufacturer's instruction. The RNA was reverse‐transcribed into cDNA using PrimeScript RT Master Mix (RR036A, Takara). Quantitative PCR was performed using TB Green Premix Ex Taq (RR420A, Takara) in a LightCyclerR480 (Roche Diagnostics Ltd., Basel, Switzerland) under identical amplification conditions. Each reaction was performed in triplicate. Target genes expression was normalized to 18S levels and quantified with the $2^{-∆∆C_{t}}$ method.

### 2.8 Enzyme‐Linked Immunosorbent Assay (ELISA)

Chemerin and ANXA1 concentration in healthy donors' and glioma patients' serum or in the 24 h supernatant of cultured GBM cells were measured by commercial Human Chemerin ELISA Kits (DCHM00, R&D Systems) and Human ANXA1 Quantikine ELISA Kit (DTA00D, R&D Systems), respectively. Optimal standard curves were applied to individual assays and the absorbance values were detected at 540 nm using a microplate reader.

### 2.9 Immunohistochemistry (IHC)

In brief, the mouse brain tumor tissues were fixed with 4% paraformaldehyde first and embedded in paraffin. Then, paraffin‐embedded tissues were cut into 4 μm sections which were incubated with the specific primary antibody against NRF2 (1:100; #ab76026, Abcam), Ki67 (1:100; #ab92742, Abcam), and ANXA1 (1:100; #ab214486, Abcam). The images were captured using an optical microscope (Olympus, Tokyo, Japan), and the German immunohistochemical method was applied to staining intensity.

### 2.10 Immunofluorescence (IF)

Briefly, the GSCs were incubated with primary antibody against NRF2 (1:100; Abcam) at 4°C overnight. Then, the rhodamine‐conjugated secondary antibody was used for multicolor immunofluorescence imaging, while DAPI solution was used for nuclear counterstaining. Finally, a laser scanning confocal microscope (Olympus) was used to visualize the staining.

### 2.11 Flow Cytometry

The cells in each experimental group underwent the following procedures: Initially, cellular digestion occurred utilizing trypsin devoid of ethylenediaminetetraacetic acid (EDTA), and subsequent collection into test tubes ensued. Subsequent centrifugation in a pre‐chilled PBS solution was conducted, followed by resuspension of the cells in 1 mL of 1× binding buffer. Annexin V, at a volume of 5 μL per tube, was introduced, and incubation transpired for 10 min at ambient temperature, shielded from light. Ultimately, staining, involving the addition of 5 μL of the stain to the tubes, was performed prior to subjecting the cells to a flow‐through assay.

### 2.12 Cell Viability Assay

Briefly, a Cell Titer 96 Aqueous Non‐Radioactive Cell Proliferation Assay Kit (Promega, Madison, WI, USA) was used to detect the cell viability according to the manufacturer's instructions. Firstly, seed the GSCs into 96‐well plates at a density of 1 × 10^3^ cells/well in triplicate. Then, the GSCs were incubated for 0, 24, 48, 72, 96, and 120 h.

### 2.13 Neurosphere Formation Assay

Briefly, seed the GSCs in 24‐well plates under the condition of 200 cells/well in a fresh medium for 7 days. Next, observe the relative neurosphere size via the optical microscope (Olympus).

### 2.14 Limiting Dilution Assay

The GSCs were seeded in 96‐well plates under different condition of 1, 10, 20, 30, 40, or 50 cells/well, meanwhile each density was replicated for 10 times. After incubation for 7 days, count the neurospheres number and calculate the neurosphere formation efficiency via the Extreme Limiting Dilution Analysis (http://bioinf.wehi.edu.au/software/elda).

### 2.15 5‐Ethynyl‐20‐Deoxyuridine (EDU) Assay

After incubation with EDU reagent for 2 h, cells were fixed with 4% paraformaldehyde (solarbio) and permeabilized with 0.5% Triton X‐100 (solarbio). The cells were then counterstained according to the production instructions of the EDU assay kit (Beyotime, Biotechnology, China). Images were captured using a laser scanning confocal microscope (Olympus) and the percentage of EDU‐positive cells was calculated.

### 2.16 Cell Migration Assay

Transwell inserts with a pore size of 8 μm (Corning, 3422) were used for cell migration in vitro. Top chambers were filled with 0.2 mL of THP1‐derived macrophage single cells (2 × 10^5^ cells per mL) in 0.2% FBS medium. The lower chamber was filled with 0.5 mL of GSC cells treated with different conditions (5 × 10^5^ cells per mL) in the configured GSC medium. After incubation for 22–24 h, the chamber membrane was fixed with methanol and stained with 0.1% crystal violet solution. The cells that had migrated to the lower side of the membrane were observed and photographed by the upright microscope. The number of cells was counted under five different random high‐power fields in each well.

### 2.17 Lipid Peroxidation (MDA) Assay

The concentration of MDA in GSC cells was assayed using the MDA assay kit (Beyotime) as per product instructions. Briefly, cells were dissociated with lysis buffer (Beyotime) and centrifuged at 12,000 *g* for 10 min at 4°C. The supernatants were collected as test samples for protein quantification using the BCA protein assay (Beyotime). Both MDA standards and test samples were added to 200 μL of TBA solution and incubated for 15 min at 100°C. After centrifugation at 1000 *g* for 10 min, the supernatants were added to a 96‐well plate and the absorbance was monitored at 531 nm. After calculating the MDA content in the test samples, the concentration of MDA in the samples was expressed as protein content per unit weight (μmol/g).

### 2.18 Reduced Glutathione (GSH) Assay

Multiple sets of glioma stem cell spheroids were collected and digested into single cells using trypsin. Digestion was terminated using FBS DMEM‐containing high‐sugar medium, washed three times with pre‐cooled PBS, and cell counting was performed. Using the GSH assay kit (Solarbio), 200 μL of kit reagent I was added at the rate of 200 μL per 10^6^ cells, sonicated in an ice bath (sonication for 3 s, repeated 30 times), followed by centrifugation for 15 min at 15,000 rpm in a centrifuge at 4°C, and the supernatant was carefully aspirated and transferred to a new centrifuge tube, which was placed on ice for later use. Dilute the standard according to 25 μg/mL, 50 μg/mL, 100 μg/mL, 150 μg/mL, 200 μg/mL, 300 μg/mL and fix 1 mL. A 1:3.5 volume of reagent 2 and reagent 3 of the kit was prepared for use. Add 20 μL of sample, 20 μL of different concentrations of standard, 20 μL of ddH_2_O in a 96‐well plate respectively, and set up three duplicate wells in each group. Add 180 μL of the mixture in the fourth step, shake and mix for 2 min, and test on the machine.

### 2.19 RNA Immunoprecipitation Assay (RIP)

RNA‐Binding Protein Immunoprecipitation Kit (Millipore) was used to perform the RIP assay according to the manufacturer's protocol. Approximately 1 × 10^7^ GSC cells under different conditions were collected respectively and then lysed in 100 μL of prepared RIP lysis buffer. The cell lysates were incubated with magnetic beads conjugated with normal rabbit IgG or U2AF65 antibodies at 4°C overnight. Subsequently, the immunoprecipitated RNAs were isolated and examined by qRT‐PCR.

### 2.20 RNA Pull‐Down Assay

Multiple sets of glioma stem cell spheroids were collected and digested into single cells using trypsin; digestion was terminated using FBS DMEM‐containing high‐sugar medium, washed three times with pre‐cooled PBS, and cell counting was performed. Add 1 mL pre‐cooled Capture Buffer (1×), 10 μL RNase inhibitor, 10 μL PMSF according to every 10^7^ cells. Centrifuge at 15,000 rpm at 4°C for 15 min and take the supernatant. Prepare the magnetic beads according to the kit instructions (Geneseed). Take 75 pmol circMAN1A2‐wt, circMAN1A2‐mt, anti‐sense, and scramble probe respectively and add 500 μL magnetic beads, and react for 30 min at 4°C in a mixer. Place in a magnetic rack and discard the supernatant. Add 500 μL Capture Buffer (1×) respectively, vortex wash for 5 s, and place in the magnetic rack. Add 500 μL Cell Lysate, place in a mixer at 10 rpm at 4°C overnight. Place on a magnetic rack, discard supernatant, and wash 3 times using Wash Buffer vortex. Add 50 μL of 4× Loading Buffer and mix, heat at 100°C in a metal bath for 5 min, and use directly for western blot to detect proteins.

### 2.21 Chromatin Immunoprecipitation Assay (ChIP)

By using the ChIP Assay Kit (Beyotime Biotechnology), the chromatin complexes were immunoprecipitated with different antibodies, respectively, according to the manufacturer's protocol. qPCR was used to determine purified DNA from the ChIP assay.

### 2.22 Luciferase Reporter Assay

The luciferase reporter plasmid (wild or mutant type of ANXA1) were constructed by Genechem (Shanghai, China). The mutant type of ANXA1 contained two mutant sites (#1 and #2) according to the prediction of Jaspar database. 5 × 10^4^ GSC cells/well were plated into 48‐well plates and cultured to 70% confluence, and the luciferase reporter plasmids were then co‐transfected into GSC for 48 h. Relative luciferase activity was determined using the Dual‐Luciferase Reporter Assay System (Promega).

### 2.23 Liquid Chromatography‐Mass Spectrometry (LC–MS/MS)

Mass spectrometry is performed according to the manufacturer's protocol. Briefly, proteins were digested into peptides with protease after being treated with a biotin‐labeled circMAN1A2 probe. Enzymatically digested peptide samples were dissolved in Nano‐LC mobile phase A (0.1% formic acid) and separated by an ultra‐high performance liquid chromatography system Easy‐nLC 2000 (ThermoFisher, USA). Peptides were analyzed by mass spectrometry on a Q Exactive mass spectrometer (Thermo Fisher Scientific, USA) equipped with a Nanospray Flex ion source (Thermo Fisher Scientific, USA). LC–MS/MS raw data were analyzed using PEAKS Studio 8.5 (Bioinformatics Solutions Inc. software., Waterloo, Canada) software for processing and retrieval, and the database was downloaded from the *Homo sapiens* protein database from Uniprot. The mass error was set at 10 ppm for precursor ions and 0.05 Da for fragment ions.

### 2.24 Intracranial Tumor Formation in Nude Mice

The experimental protocol was reviewed and approved by the Laboratory Animal Ethics Committee of China Medical University (KT2022436), and the experiments were performed in the SPF laboratory. The mice used in the experiment were female. GL261 cells were collected from each group, digested, and processed into a single cell suspension using trypsin, centrifuged at 1000 rpm for 5 min, the supernatant discarded, and placed on ice for use. C57 mice aged 4–6 weeks were selected and anesthetized by injection of 0.75% sodium pentobarbital in the left lower abdomen, and fixed on the stereotaxic apparatus after successful anesthesia. The heads of nude mice were sterilized using alcohol, and the skin was incised bluntly separated, and isolated in the midline. A needle was inserted 2 mm to the right of the parallel fontanel to slowly inject 3 μL of cytosolic precipitates, which were completely injected and then left for 2 min to exit slowly. Bone wax was closed and the skin was sutured.

### 2.25 Statistical Analysis

All experiments underwent at least three independent replications and were presented using mean ± standard deviation. Statistical tests were performed using chi‐square test, *t*‐test, one‐way ANOVA, and the Wilcoxon rank sum test. Pearson correlation coefficient was used to detect genetic correlation. Survival differences were assessed using the log‐rank test and Kaplan–Meier. *p* < 0.05 was considered statistically significant. R version 4.1.3 or GraphPad Prism 8.0.2 were used for all relevant statistical analyses.

## 3 Result

### 3.1 CircMAN1A2 Expression Is Downregulated in Glioma and Indicates Poor Prognosis

**FIGURE 1:**
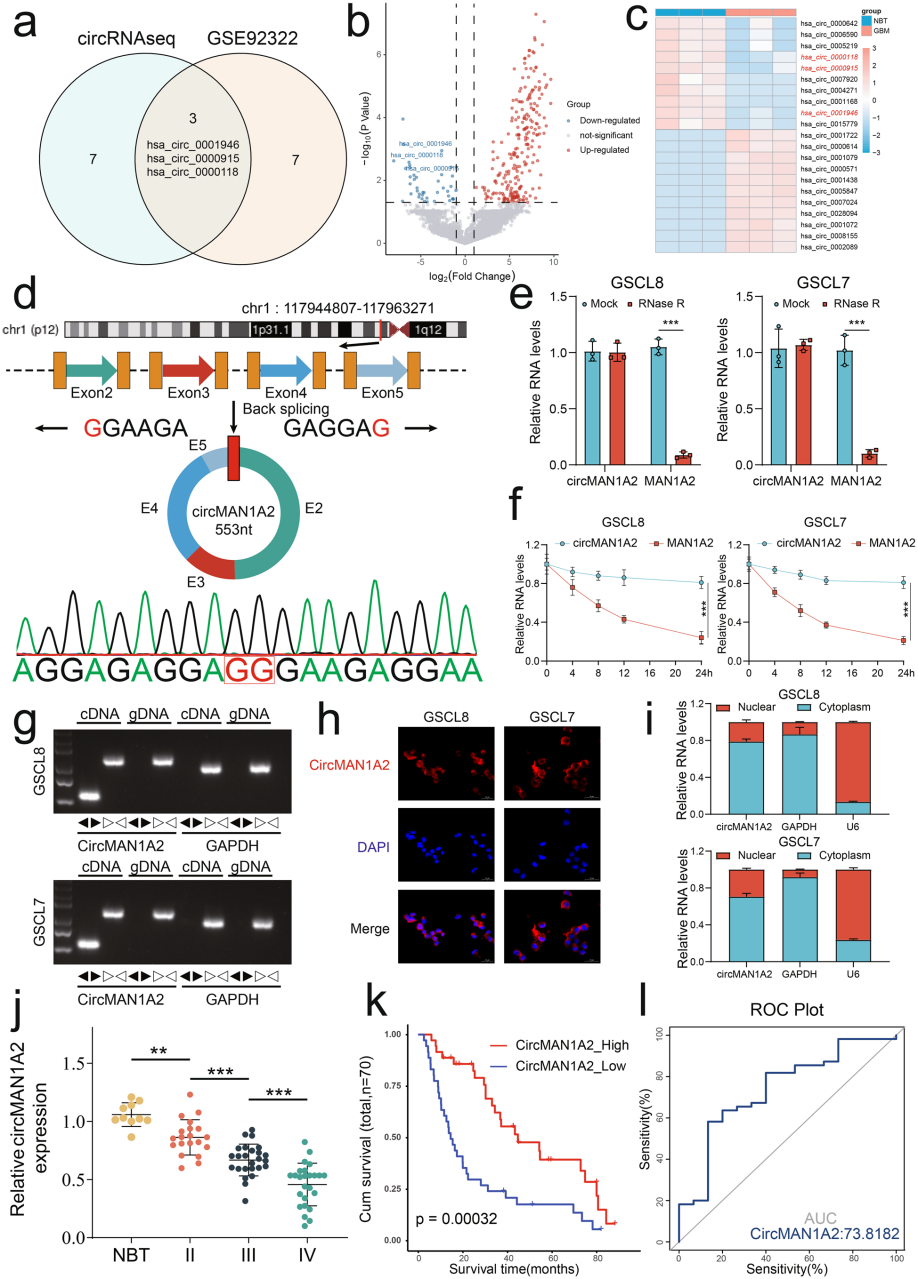
CircMAN1A2 expression is downregulated in glioma and indicates poor prognosis. (a) Venn diagram delineating the exclusive and shared circRNAs between the circRNAseq and GSE92322 datasets, pinpointing three circRNAs unique to the circRNAseq database. (b) Volcano plot categorizing the circRNAs based on their expression levels in glioma; red dots signify upregulation, blue dots indicate downregulation, and gray dots represent non‐significant differences. The negative logarithm of the *p*‐value (*y*‐axis) is plotted against the log2‐transformed fold change (*x*‐axis), with circMAN1A2 notably highlighted. (c) Heatmap displaying expression profiles of top differentially expressed circRNAs in glioma versus control brain matter (CBM). Intensity of color corresponds to expression magnitude, with red indicating high expression and blue indicating low expression. (d) Schematic representation of the genomic locus of circMAN1A2, illustrating its formation through back‐splicing of exons 2 to 5 of the MAN1A2 gene, accompanied by the sequence of the back‐splicing junction (displayed as a green waveform). (e) Quantitative bar graphs comparing the relative RNA levels of circMAN1A2 and linear MAN1A2 post‐RNase R treatment, demonstrating the nuclease resistance and circular nature of circMAN1A2 as evidenced by the significant maintenance of its levels (***p < 0.001). (f) Line graphs depicting the temporal decline in circMAN1A2 and MAN1A2 RNA levels following siRNA‐mediated knockdown in GSCL8 and GSCL7 cell lines, validating the knockdown's specificity and efficiency. (g) Agarose gel electrophoresis validating the amplification of circMAN1A2 from cDNA but not genomic DNA (gDNA), thereby confirming its circular RNA identity. (h) Fluorescent in situ hybridization (FISH) images showing the subcellular localization of circMAN1A2 (red signals) within GSCL8 and GSCL7 cells, with nuclear staining by DAPI (blue). (i) Nucleoplasmic expression of circMAN1A2 in GSCL8 and GSCL7. (j) Expression of circMAN1A2 in NBT and different WHO grade glioma samples. (NBT, *n* = 10; WHO grade II, *n* = 20; WHO grade III, *n* = 25; WHO grade IV, *n* = 25). (k) Kaplan–Meier analysis was performed on 70 patients with gliomas to determine the relationship between circMAN1A2 expression levels and overall survival. (l) ROC curves of circMAN1A2 expression in 70 glioma patients. All results are expressed as SD ± mean (three independent experiments). *p < 0.05, **p < 0.01, ***p < 0.001, ****p < 0.0001.

To identify circRNAs involved in glioma progression, we performed circRNA sequencing of clinical samples and adjacent tissues from three glioblastoma patients along with bioinformatics analysis. We further obtained and analyzed the public dataset GSE92322 containing samples from 5 GBM pairs and adjacent tissues. Cross‐analysis of the top 10 downregulated circRNAs identified in both our sequencing and GSE92322 revealed three shared downregulated circRNAs (hsa_circ_0001946, hsa_circ_0000915, hsa_circ_0000118) in GBM patients (Figure 1a–c). We then determined the expression of these three selected circRNAs in GBM tissues using RT‐qPCR. The results showed that hsa_circ_0000118 exhibited the most significant downregulation compared to the other two candidates (Figure S1a). Therefore, we selected hsa_circ_0000118 (hereafter referred to as circMAN1A2) for further investigation. Our data indicated that circMAN1A2 was derived from the *mannosidase alpha class 1A member 2(MAN1A2)* gene and formed by back‐splicing from exon 2 to exon 5 (Figure 1d). We next determined circMAN1A2 expression in patient‐derived GSCs cell lines (Figure S1b). The results showed that circMAN1A2 expression levels were lower in GSCL8 and GSCL3 cells and higher in GSCL9 and GSCL7 cells. On the basis of these findings, we selected GSCL8 and GSCL7 to further validate the circular RNA characterization of circMAN1A2 and its cellular distribution. Sanger sequencing confirmed the reverse splicing junction site of circMAN1A2 (Figure 1d). RNase R treatment and actinomycin D treatment assays demonstrated that circMAN1A2 was resistant to RNase R digestion and exhibited a longer half‐life compared to linear MAN1A2 mRNA, confirming its high stability (Figure 1e,f). Agarose gel electrophoresis of qPCR products further revealed that the circMAN1A2 divergent primers amplified a product only from cDNA, not from genomic DNA (Figure 1g). We then investigated the cellular localization of circMAN1A2 using cytoplasmic isolation and RNA FISH. These experiments demonstrated that circMAN1A2 was predominantly localized in the cytoplasm (Figure 1h,i). Moreover, we examined circMAN1A2 expression levels in 70 clinical specimens (10 adjacent tissues, 20 WHO grade II, 25 grade III, and 25 grade IV) using RT‐qPCR. CircMAN1A2 expression levels were significantly decreased in glioma samples and exhibited a significant negative correlation with the WHO grade of gliomas, with the most significant reduction observed in grade IV glioma samples (Figure 1j). Kaplan–Meier survival analysis of patient subgroups categorized based on the median circMAN1A2 expression value (circMAN1A2_High and circMAN1A2_Low) from the clinical samples revealed that lower circMAN1A2 expression was significantly associated with a poorer prognosis in glioma patients (Figure 1k). The receiver operating characteristic (ROC) curve analysis yielded an area under the curve (AUC) value of 0.738, indicating that circMAN1A2 could serve as a potential biomarker for predicting the survival prognosis of glioma patients (Figure 1l). In conclusion, we identified circMAN1A2 as a novel circRNA that is downregulated in glioma and exhibits a negative correlation with poor patient prognosis.

### 3.2 circMAN1A2 Plays an Inhibitory Role in the Malignant Progression of GSCs

**FIGURE 2:**
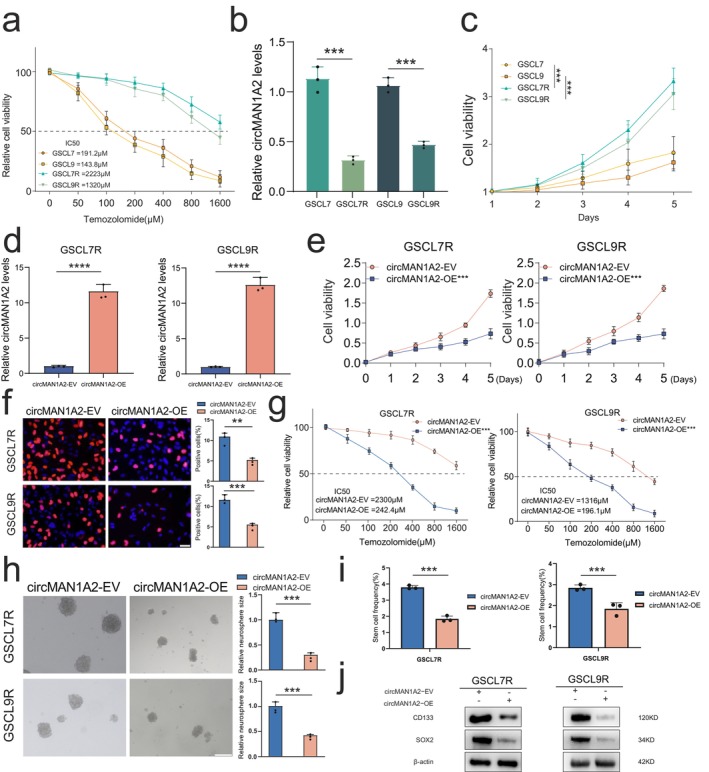
circMAN1A2 plays an inhibitory role in the malignant progression of GSCs. (a) The IC_50_ of GSCL9 and GSCL7 normal cell lines against TMZ was calculated using CCK8. (GSCL7 = 191.2 μM, GSCL9 = 143.8 μM). TMZ‐resistant cell lines GSCL7R and GSCL9R were constructed and the corresponding IC_50_s were calculated using CCK8 (GSCL7R = 2223 μM, GSCL9R = 1320 μM). (b) MTS assay for cell viability of normal cell lines of GSCL9 and GSCL7 as well as TMZ‐resistant cell lines (***p < 0.001). (c) qRT‐PCR validation of mRNA expression levels of circMAN1A2 in normal versus TMZ‐resistant cell lines of GSCL7 (left) and GSCL9 (right). (d) Transfection efficiency of overexpressed circMAN1A2 in GSCL7R (left) and GSCL9R (right) was detected by qPCR (****p < 0.0001). (e) MTS assay for cell viability of GSCL7R (left) and GSCL9R (right) overexpressing circMAN1A2 (***p < 0.001). (f) CCK8 calculated IC_50_ for TMZ after overexpression of circMAN1A2 in GSCL7R (left) and GSCL9R (right) (***p < 0.001). EDU (g) neurosphere formation assay (h) and limiting dilution assay (i) to detect malignant phenotypes in GSCL7R (upper) and GSCL9R (lower) cell lines after overexpression of circMAN1A2. (j) Western blot revealed the extent of stemness indicator expression in GSCL7R (left) and GSCL9R (right) cell lines after overexpression of circMAN1A2. All results are expressed as SD ± mean (three independent experiments). *p < 0.05, **p < 0.01, ***p < 0.001, ****p < 0.0001.

To investigate the biological function of circMAN1A2 in GSCs, we selected GSC cell lines GSCL7 and GSCL9 with higher circMAN1A2 expression for circMAN1A2 knockdown and GSCL3 and GSCL8 for circMAN1A2 overexpression. qPCR results confirmed that circMAN1A2‐KD1, circMAN1A2‐KD2, and circMAN1A2‐OE significantly altered circMAN1A2 expression levels compared to the control groups (Figure S2a,b). We examined cell proliferation using 3D cell viability and EdU assays. The results showed that circMAN1A2 knockdown promoted the proliferation of GSCL7 and GSCL9 cells, while circMAN1A2 overexpression inhibited the proliferation of GSCL3 and GSCL8 cells (Figure S2c–f). Next, we assessed the effects of circMAN1A2 on GSC stemness using neurosphere formation assays and the extreme limiting dilution assay. The results demonstrated that circMAN1A2 overexpression significantly decreased GSCs sphere formation capacity, while knockdown of circMAN1A2 increased sphere formation (Figure S2g–j). In addition, the western blot of GSC stem marker protein CD133 and SOX2 showed similar results. Compared with the control group, the knockdown of circMAN1A2 significantly enhanced the invasive ability of GSCL7 cells but significantly reduced the invasive ability of GSCL3 overexpressing cells (Figure S2k). Considering the critical role of GSCs in glioma recurrence and TMZ resistance [25, 26], we established TMZ‐resistant GSC cell lines, GSC7R and GSLC9R, derived from GSCL7 and GSCL9 cells with higher circMAN1A2 expression. Cell viability assays showed that the IC_50_ (half maximal inhibitory concentration) of TMZ in GSCL7R and GSLC9R cells was significantly higher compared to GSCL7 and GSCL9 cells (Figure 2a). RT‐qPCR analysis revealed that circMAN1A2 expression was decreased in the TMZ‐resistant cell lines, GSC7R and GSCL9R (Figure 2b). Further cell viability assays confirmed that TMZ‐resistant cell lines exhibited greater proliferative activity than the original GSC cell lines (Figure 2c). These results suggest that circMAN1A2 may play a role in the TMZ resistance of GSCs. We next overexpressed circMAN1A2 to explore its function in TMZ‐resistant GSCs (Figure 2d). CircMAN1A2 overexpression significantly suppressed the proliferation and stemness of these cells (Figure 2e,f,h–j) and reversed TMZ resistance in the TMZ‐resistant GSCs (Figure 2g). Our findings indicate that circMAN1A2 can inhibit malignant progression in GSCs.

### 3.3 PRPF40B Promotes circMAN1A2 Biogenesis and Inhabit Proliferation in GSCs

To investigate the upstream regulation of circMAN1A2, we analyzed the TCGA dataset to identify genes involved in circRNA biogenesis that were also downregulated in glioma. We found that PRPF40B expression inversely correlated with WHO grade and that lower PRPF40B expression connoted a poor prognosis in glioma patients (Figure S3a,b). RT‐qPCR analysis of clinical samples revealed a simultaneous increase in circMAN1A2 expression with increasing PRPF40B expression (Figure S3c). We then constructed PRPF40B knockdown (GSCL7(PRPF40B‐KD)) and overexpression (GSCL8(PRPF40B‐OE)) cell lines (Figure S3d,e). RT‐qPCR results showed that PRPF40B knockdown downregulated circMAN1A2 expression in GSCL7 cells, whereas PRPF40B overexpression significantly increased circMAN1A2 expression in GSCL8 cells (Figure S3f). 3D cell viability, EdU, and ELDA assays demonstrated that PRPF40B knockdown enhanced the proliferative viability of GSCs, which was abrogated by circMAN1A2 overexpression. Conversely, PRPF40B overexpression inhibited the proliferative viability of GSCs, and this effect was reversed by circMAN1A2 knockdown (Figure S3g–j). These results suggest that PRPF40B promotes circMAN1A2 biogenesis and suppresses the proliferation of GSCs.

### 3.4 CircMAN1A2 Causes TMZ Sensitive in GSCs via Inducing Ferroptosis

**FIGURE 3:**
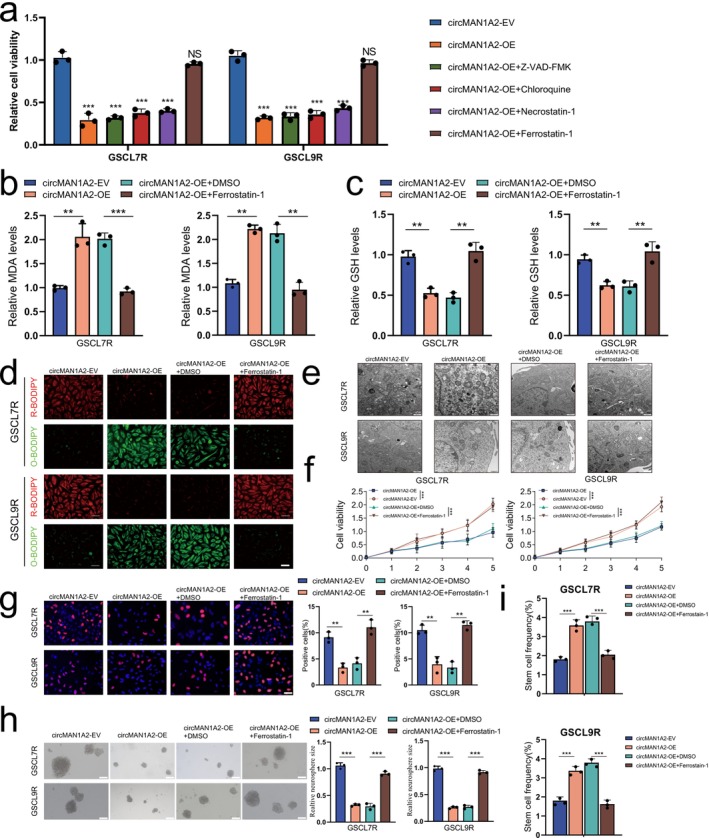
CircMAN1A2 causes TMZ sensitive in GSCs via inducing ferroptosis. (a) MTS assay for cell viability after overexpression of CircMAN1A2 in GSCL7R (left) and GSCL9R (right) cell line treated with inhibitors such as Z‐VAD‐FMK, Chloroquine, Necrostatin‐1 and Ferrostatin‐1, respectively. Only Ferrostatin‐1 could revert to proliferative activity (***p < 0.001). (b) In the GSCL7R (left) and GSCL9R (right) cell lines, we overexpressed circMAN1A2 and performed rescue experiments using Ferrostatin‐1, subsequently detecting changes in MDA expression levels. (c) In the GSCL7R (left) and GSCL9R (right) cell lines, we overexpressed circMAN1A2 and performed rescue experiments using Ferrostatin‐1, subsequently detecting changes in GSH expression levels. (d) BODIPY detection of ROS levels (green signal) in GSCL7R (left) cell line and GSCL9R (right) after different treatments. (e) Transmission electron microscopy was performed to observe the mitochondrial changes in cells after overexpression of CircMAN1A2 in GSCL9R (left) and GSCL7R (right) cell line and treatment with DMSO and Ferrostatin‐1, respectively. (f) MTS assay for cell viability after overexpression of CircMAN1A2 in GSCL9R (left) and GSCL7R (right) cell line and treatment with DMSO and Ferrostatin‐1, respectively. (g) EDU examined the cellular value‐added capacity after overexpression of CircMAN1A2 in GSCL9R (left) and GSCL7R (right) cell line and treatment with DMSO and Ferrostatin‐1, respectively. (h, i) Neurosphere formation assay and limiting dilution assay were performed to detect changes in cell stemness after overexpression of CircMAN1A2 in GSCL9R (left) and GSCL7R (right) cell line and treatment with DMSO and Ferrostatin‐1, respectively. All results are expressed as SD ± mean (three independent experiments). *p < 0.05, **p < 0.01, ***p < 0.001, ****p < 0.0001.

Programmed cell death (PCD) is essential for cellular homeostasis and plays a critical role in tumor progression. TMZ, as the standard monotherapy for GBM, has limited effectiveness due to TMZ resistance. Its primary antitumor effect is through inducing DNA damage, leading to apoptosis. However, GSCs possess resistance to apoptosis and can survive TMZ treatment [27]. Enhancing TMZ efficacy by modulating PCD has shown promising therapeutic outcomes in preclinical studies. To investigate whether circMAN1A2 is involved in programmed cell death of TMZ‐resistant GSCs, we treated the circMAN1A2‐OE cell model, GSCL7R and GSCL9R, with apoptosis inhibitor Z‐VAD‐FMK, autophagy inhibitor Chloroquine, necrosis inhibitor Necrostatin‐1, and ferroptosis inhibitor Ferrostatin‐1. 3D cell viability detection showed that only Ferrostatin‐1 treatment effectively restored cellular proliferative activity (Figure 3a). To verify whether circMAN1A2 actually affects glioma cell viability via the ferroptosis pathway, we examined changes in MDA and GSH levels after treatment by Ferrostatin‐1 in drug‐resistant cell lines overexpressing circMAN1A2. The results showed that MDA levels were elevated and GSH levels were decreased in drug‐resistant cell lines overexpressing circMAN1A2, and the expression levels of MDA and GSH reverted after treatment with the ferroptosis inhibitor Ferrostatin‐1 (Figure 3b,c). Immunofluorescence and flow cytometry analyses revealed that oxygen species (ROS) levels were elevated in TMZ‐resistant GSCs overexpressing circMAN1A2, while treatment with Ferrostatin‐1 reversed the high ROS levels induced by circMAN1A2 overexpression (Figure 3d, Figure S4). Transmission electron microscopy revealed that TMZ‐resistant GSCs with circMAN1A2 overexpression exhibited mitochondrial damage, characterized by aberrant crumpling of the mitochondrial membrane and a reduction in cristae. Ferrostatin‐1 treatment eliminated these pathological features (Figure 3e). Next, we employed 3D cell viability detection (Figure 3f), EdU (Figure 3g), neurosphere formation (Figure 3h), and ELDA assays (Figure 3i) to examine the effect of circMAN1A2‐induced ferroptosis on the malignant phenotypes of TMZ‐resistant GSCs. The results demonstrated that Ferrostatin‐1 treatment restored GSC cell viability and stemness that were inhibited by circMAN1A2 overexpression. In conclusion, the data presented above suggest that circMAN1A2 inhibits the malignant phenotypes of TMZ‐resistant GSCs by inducing ferroptosis.

### 3.5 CircMAN1A2 Can Competitively Bind to TEP1 With KEAP1 to Reduce NRF2 Expression

**FIGURE 4:**
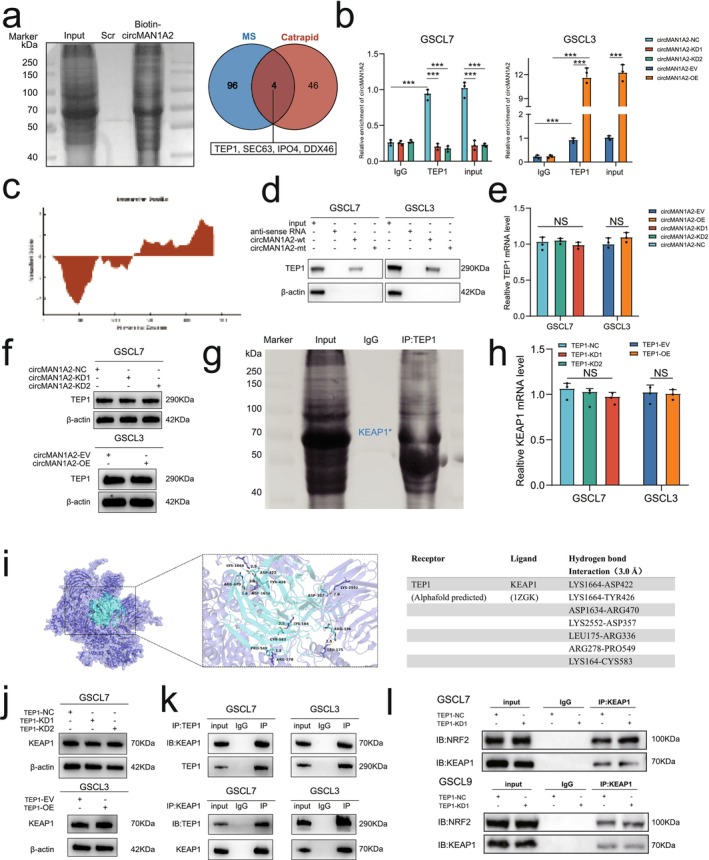
CircMAN1A2 can competitively bind to TEP1 with KEAP1 to reduce NRF2 expression. (a) Proteins interacting with circMAN1A2 were identified by LC–MS/MS and Catrapid database after traction with a biotin‐labeled circMAN1A2 probe. (b) The RIP assay showed the enrichment level of circMAN1A2 in GSCL7 (left) and GSCL9 (right) cell line. (c) The Catrapid database predicts the binding site of circMAN1A2 to TEP1. (d) RNA pulldown characterize circMAN1A2 interaction with TEP1 protein. (e) qRT‐PCR was performed to detect the mRNA expression level of TEP1 in circMAN1A2 knockdown and overexpression treatment groups, TEP1 was not significantly altered. (f) Western blot was performed to detect the protein expression level of TEP1 in circMAN1A2 knockdown and overexpression treatment groups, TEP1 was not significantly altered. (g) MS analysis of TEP1‐bound proteins, KEAP1 was most significantly reduced. (h) qRT‐PCR was performed to detect the mRNA expression level of KEAP1 in circMAN1A2 knock‐down and overexpression treatment groups. (i) Bioinformatic analysis predicted that TEP1 contains multiple binding sites within the conserved Kelch domain of KEAP1 (amino acids 322‐609), the region responsible for NRF2 binding. (j) Western blotting was used to assess KEAP1 protein levels in TEP1 knock‐down and overexpression treatment groups. (k) Co‐IP assays confirmed that TEP1 can interact with KEAP1. (l) Co‐IP analysis was conducted to evaluate changes in KEAP1–NRF2 binding in TEP1 knock‐down versus control groups. All results are expressed as SD ± mean (three independent experiments). *p < 0.05, **p < 0.01, ***p < 0.001, ****p < 0.0001.

Previous studies have reported that circRNAs can regulate the biological functions of related proteins through direct binding. To explore the downstream mechanisms of circMAN1A2, we used the bioinformatics tool CatRAPID to predict proteins that may interact with circMAN1A2. By integrating the top 100 predicted proteins from CatRAPID with the top 50 proteins identified through RNA‐pulldown combined with LC–MS/MS analysis, we obtained four candidate proteins (TEP1, SEC63, IPO4, DDX46) (Figure 4a). Among these four proteins, TEP1 stood out, given its highest score in the mass spectrometry data (‐10lgP = 299.87) and its highest binding intensity score in the CatRAPID prediction results (91.08) (Tables S2 and S3). Additionally, analysis of TCGA, CGGA, and Rembrandt datasets revealed that high levels of TEP1 expression predicted a poor prognosis for patients (Figure S5a). Therefore, TEP1 was selected for further investigation. We next validated the binding of circMAN1A2 to TEP1 in GSCL7 and GSCL3 cells. RIP assays demonstrated significant enrichment of circMAN1A2 in the anti‐TEP1 group compared to the anti‐IgG group. Furthermore, the enrichment levels of circMAN1A2 were significantly decreased in GSCL7 cells following circMAN1A2 knockdown, whereas circMAN1A2 enrichment levels were increased in GSCL3 cells upon circMAN1A2 overexpression (Figure 4b). As predicted by the CatRAPID database, multiple regions of the TEP1 protein could bind to the 326–377 region of circMAN1A2 (Figure 4c). The RNA pull‐down assay further confirmed our findings. The biotinylated wild‐type circMAN1A2 probe successfully pulled down the TEP1 protein in both GSCL7 and GSCL3 cells, while the circMAN1A2‐mt probe did not (Figure 4d). These results collectively indicate that circMAN1A2 interacts with the TEP1 protein.

To investigate whether TEP1 is a downstream effector of circMAN1A2, we further examined its expression level. The results of RT‐qPCR indicated that altering circMAN1A2 expression did not change the transcript level of TEP1 (Figure 4e). The results of Western blot also indicated that TEP1 was not affected by circMAN1A2 expression at the protein level (Figure 4f). However, functional assays demonstrated that TEP1 overexpression restored the decreased cell proliferation and stemness and suppressed ferroptosis observed in TMZ‐resistant cell lines GSCL9R and GSCL7R upon circMAN1A2 overexpression (Figure S5b–i). On the basis of these findings, we hypothesized that circMAN1A2 exerts its biological function by affecting the interaction between TEP1 and other proteins. We then performed a co‐immunoprecipitation assay with TEP1, followed by mass spectrometry analysis to identify TEP1‐interacting proteins. Interestingly, the analysis identified KEAP1, a protein known to be closely linked to ferroptosis regulation (Figure 4g). Both RT‐qPCR and Western blot analyses revealed that TEP1 expression did not affect KEAP1 protein levels (Figure 4h,j). KEAP1 is an E3 ubiquitin ligase that normally forms a complex with NRF2, a key regulator of the antioxidant response, to regulate NRF2 protein levels. Under conditions of oxidative stress, such as ferroptosis, KEAP1 releases NRF2 to maintain cellular homeostasis [28]. In silico docking analysis predicted a strong binding affinity between TEP1 and KEAP1, with a binding score of −718. Additionally, TEP1 was predicted to have multiple binding sites within the conserved Kelch domain (amino acids 322–609) of KEAP1, the region responsible for NRF2 binding (Figure 4i). A co‐immunoprecipitation (Co‐IP) assay confirmed that TEP1 could interact with KEAP1 (Figure 4k). Moreover, TEP1 knockdown enhanced the interaction between NRF2 and KEAP1 (Figure 4l). These findings suggest that circMAN1A2 promotes KEAP1‐NRF2 binding by competitively binding to TEP1. It is worth noting that, although we have focused on GSCs, considering the many differences in expression between GSCs and non‐GSCs, the regulation of the circMAN1A2 axis in non‐GSCs should also be studied. Therefore, we conducted additional experiments using differentiated cells derived from GSCs. Our results showed that while similar changes were observed in the differentiated cell lines, these regulatory effects appeared to be less pronounced compared to those observed in GSCs (Figure S6a–d). The regulatory effect of circMAN1A2 does not appear to be limited to GSCs alone.

### 3.6 TEP1 Competitively Binds KEAP1 to NRF2 via ESGE Motifs to Promote NRF2 Expression and Nuclear Translocation

**FIGURE 5:**
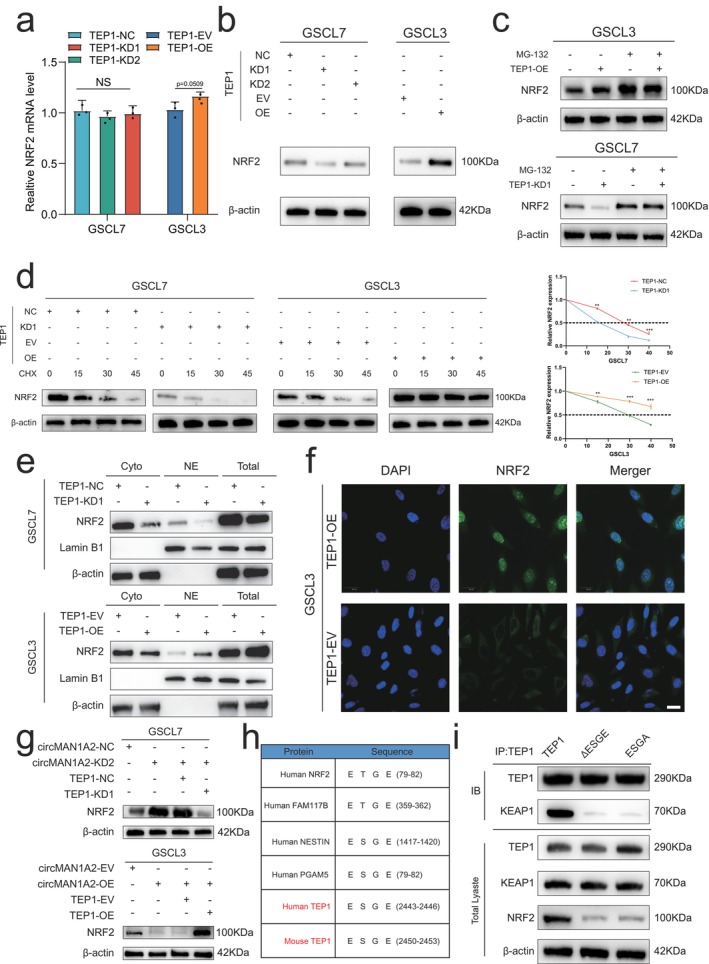
TEP1 competitively binds KEAP1 to NRF2 via ESGE motif to promote NRF2 expression and nuclear translocation. (a) qRT‐PCR was performed to detect changes in the mRNA expression levels of NRF2 in GSC cell lines treated with TEP1 knockdown and overexpression. (b) Western blot detection of protein changes of NRF2 in GSC cell lines treated with TEP1 knockdown and overexpression. (c) Western blotting showed NRF2 protein levels in TEP1 knockdown or overexpressed GSCs treated with MG132 (50 μM) for 6 h. (d) Protein levels of NRF2 were detected by Western blotting in cycloheximide (CHX, 100 ng/mL)‐treated GSC knocked down or overexpressing TEP1. (e) Western blotting was performed to detect the levels and ratios of NRF2 proteins in nuclear and cytoplasmic lysates of GSC cells knocking down or overexpressing TEP1. (f) Immunofluorescence detection of NRF2 protein distribution in TEP1 overexpressing GSCs. Scale bar = 50 μM. (g) Western blotting was performed to detect changes in the expression level of NRF2 protein in circMAN1A2 knockdown TEP1 overexpression and circMAN12 overexpression TEP1 knockdown GSC cell lines, respectively. (h) NRF2 protein expression and binding to KEAP1 after deletion or mutation of the ESGE motif of TEP1. (i) Protein sequence localization of EST/GE motifs.

NRF2 is a key transcription factor that helps cells resist oxidative stress in vivo. It regulates the level of ferroptosis, and its protein level is tightly controlled by KEAP1 [29, 30]. We found that TEP1 competitively binds KEAP1 and predicted that TEP1 and KEAP1 share multiple potential binding sites within the NRF2‐binding Kelch domain. To investigate whether TEP1 regulates NRF2 expression by binding to KEAP1, we performed RT‐qPCR assays. These revealed negligible changes in NRF2 mRNA levels in glioma cell lines following TEP1 knockdown or overexpression (Figure 5a). However, Western blot analysis showed a decrease in NRF2 protein levels following TEP1 knockdown in GSCL7 cells, whereas TEP1 overexpression led to an increase in NRF2 protein levels in GSCL3 cells (Figure 5b). These results suggest that TEP1 primarily regulates NRF2 protein expression, not NRF2 mRNA expression. Further experiments demonstrated that the proteasome inhibitor MG‐132 could reverse the decrease in NRF2 protein levels after TEP1 knockdown in GSCL7 cells and increased NRF2 protein levels in GSCL3 cells with TEP1 overexpression following MG‐132 treatment (Figure 5c). Similarly, treatment with the translation inhibitor cycloheximide (CHX) reduced NRF2 protein half‐life in the TEP1 knockout group, while it yielded the opposite effect in the TEP1 overexpression group (Figure 5d). We next assessed the distribution of NRF2 in GSCs by Western blotting and immunofluorescence. The results showed that TEP1 overexpression increased the total and nuclear expression levels of NRF2 protein while decreasing the cytoplasmic expression level of NRF2 protein. Conversely, TEP1 knockdown resulted in the opposite effect (Figure 5e,f). Moreover, circMAN1A2 knockdown elevated NRF2 protein expression levels, which was counteracted by TEP1 knockdown, resulting in decreased NRF2 levels in GSCL7 cells. Contrasting results were observed in the circMAN1A2‐overexpressing cell line GSCL3 (Figure 5g). Collectively, these findings demonstrate that circMAN1A2 does not directly alter mRNA or protein levels of TEP1. Instead, it functions as a tumor suppressor by competitively binding to TEP1. By analyzing the TEP1 protein sequence, we identified an ESGE motif known to bind KEAP1. CO‐IP results showed that deletion or mutation of the ESGE motif in TEP1 reduced its binding to KEAP1, which in turn inhibited NRF2 protein levels (Figure 5h,i). Additionally, considering that KEAP1 is an E3 ubiquitin ligase, we examined the ubiquitination levels of NRF2. The results indicated that the deletion or mutation of the ESGE motif in TEP1 reduced the ubiquitination levels of NRF2 (Figure S7). In summary, the results presented above suggest that TEP1 relies on its ESGE sequence to competitively bind KEAP1 and promote NRF2 protein expression and nuclear localization, thereby contributing to the ferroptosis resistance observed in TMZ‐resistant GSCs. CircMAN1A2 disrupts TEP1 binding to KEAP1, leading to NRF2 degradation and ultimately inducing ferroptosis.

### 3.7 NRF2 Transcriptionally Upregulates ANXA1 Expression in GSC to Promote Tumor‐Associated Macrophage Recruitment and M2 Polarization

**FIGURE 6:**
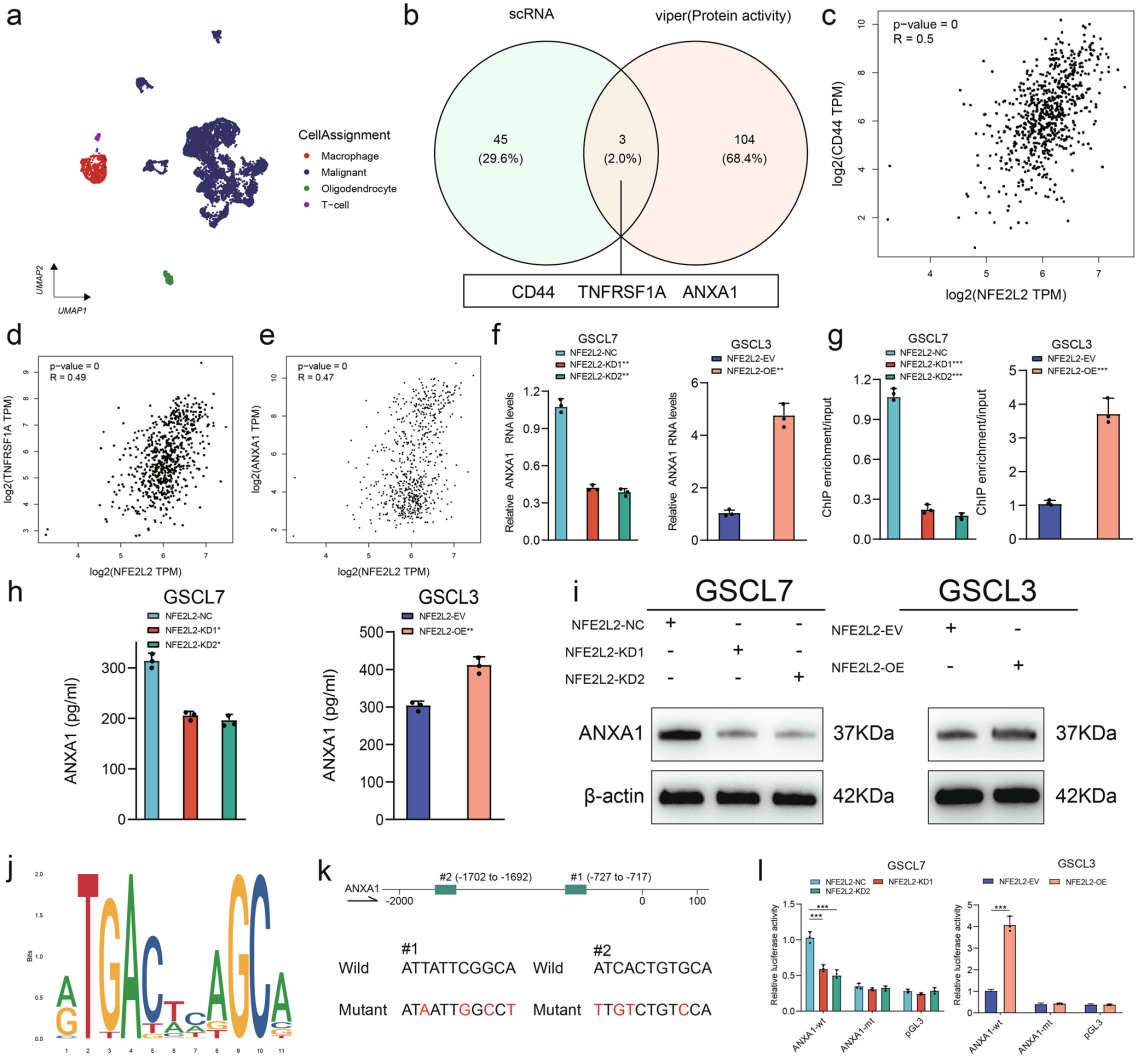
NRF2 transcriptionally upregulates ANXA1 expression in GSC to promote TAM recruitment and M2 polarization. (a) The umap plot of GSE131928 single cell sequencing. (b) Venn diagram of the intersection of differentially transcribed genes and genes differentially upregulated for protein activity. (c–e) Correlation analysis of NFE2L2 with CD44, TNFRSF1A, ANXA1 in glioma tissues (data from GEPIA2 database). (f) The qPCR results showed the level of ANXA1 mRNA expression in GSC that interfered with NFE2L2. (g) ChIP assay showing the level of ANXA1 transcriptional promoter enrichment after interference with NFE2L2. (h) ELISA assay showing the expression level of ANXA1 protein in cell supernatants after interference with NFE2L2. (i) Western blot assay showing the expression level of ANXA1 protein in cells after interference with NFE2L2. (j) Schematic representation of the DNA binding motif of NFE2L2. (k) Schematic representation of the ANXA1 promoter mutation. (l) Dual luciferase reporter gene assay interferes with ANXA1 transcriptional activity after NFE2L2.

Current studies on the role of NRF2 in immune regulation are primarily based on the function of immune cells themselves. In gliomas, research has found that NRF2 gene expression is associated with macrophage infiltration in low‐grade gliomas [31]. Therefore, we focused on the molecular mechanisms by which the NRF2 regulatory network may be involved in the GSC‐TAM interaction network. Transcription factors are key regulators of numerous genes that control cell types, developmental patterns, and specific cellular programs [32]. NRF2, for instance, has been shown to recruit macrophages toward wounds in keratinocyte cells by upregulating the expression of the secreted protein CCL2 [33]. By analogy, we are attempting to identify transcription factors that NRF2 might regulate in GBM. To identify potential ligand‐receptor proteins regulated downstream of NRF2, we analyzed GBM single‐cell sequencing data (GSE131928) from 28 patients with IDH‐wild type tumors. This data included 7930 cells (comprising 6863 tumor cells, 754 macrophages, 219 oligodendrocytes, and 94T cells) profiled using Smartseq2 sequencing technology (Figure 6a). We then calculated the activity of NRF2 and ligand‐receptor proteins in each cell. Subsequently, we performed a cross‐analysis to identify ligand‐receptor proteins that were upregulated at both the RNA and protein activity levels in tumor cells exhibiting high NRF2 protein activity. RNA levels and protein activity of CD44, TNF Receptor Superfamily Member 1A (TNFRSF1A), and ANXA1 were elevated in these high NRF2 tumor cells (Figure 6b). TCGA database analysis further revealed a strong positive correlation between NFE2L2 (the gene encoding NRF2) and CD44, TNFRSF1A, and ANXA1 expression in glioma tissues (Figure 6c–e). Considering that CD44 and TNFRSF1A primarily function as receptors, while ANXA1 can participate in regulating the tumor immune microenvironment as a secreted protein [34], we focused on whether NRF2 regulates the tumor immune microenvironment through ANXA1. Analysis of TCGA, CGGA, and Rembrandt datasets revealed that high ANXA1 expression predicted poor prognosis in glioma patients (Figure S8a–c). We then manipulated NFE2L2 expression levels. qRT‐PCR analysis showed that ANXA1 gene expression levels were significantly suppressed in the NFE2L2 knockdown cell line GSCL7 and significantly upregulated in the NFE2L2 overexpressing cell line GSCL3 (Figure 6f). A ChIP assay confirmed these findings, demonstrating that the enrichment level of ANXA1 was significantly increased in the NFE2L2 overexpression group and decreased in the NFE2L2 knockdown group (Figure 6g). ELISA and Western blot assays yielded similar results, with ANXA1 protein expression downregulated and suppressed by NFE2L2 knockdown in GSCL7 and, conversely, increased by NFE2L2 overexpression in GSCL3 (Figure 6h,i). We next performed a co‐culture experiment between GSC cells and macrophages (Figure S9a). TAM recruitment assays revealed that NFE2L2 knockdown in GSCL7 cells inhibited TAM recruitment. Interestingly, overexpression of ANXA1 or treatment with recombinant ANXA1 protein significantly restored TAM recruitment. Conversely, NFE2L2 overexpression in GSCL3 cells resulted in the opposite effect (Figure S9b). Furthermore, culturing TAMs using GSC supernatants demonstrated that NFE2L2 overexpression strongly upregulated the expression of M2 marker genes (CD206, Arg1, and IL10) in TAMs. This upregulation was significantly inhibited by the knockdown of ANXA1 or treatment with an ANXA1 antibody. In contrast, NFE2L2 knockdown inhibited the expression of CD206, Arg1, and IL10 in TAMs, while overexpression of ANXA1 or treatment with recombinant ANXA1 protein upregulated the expression of these M2 marker genes (Figure S9c), Flow cytometry analysis also validated this result (Figure S9d). Finally, we utilized the Jaspar database to predict NRF2 transcriptional binding sites within ANXA1 and constructed ANXA1 mutant luciferase reporter genes based on these predictions (Figure 6j,k). Luciferase reporter assays indicated that silencing NRF2 reduced the luciferase activity of the wild‐type ANXA1 reporter (ANXA1‐wt), whereas NRF2 overexpression increased the activity of ANXA1‐wt. Conversely, the luciferase activity of ANXA1‐mt was not affected by changes in NRF2 (Figure 6l). Altogether, our results showed that the upregulation of ANXA1 by NRF2 promotes the recruitment and M2 polarization of tumor‐associated macrophages.

### 3.8 CircMAN1A2 Suppresses Glioma Progression and Promotes TMZ Sensitive In Vivo

**FIGURE 7:**
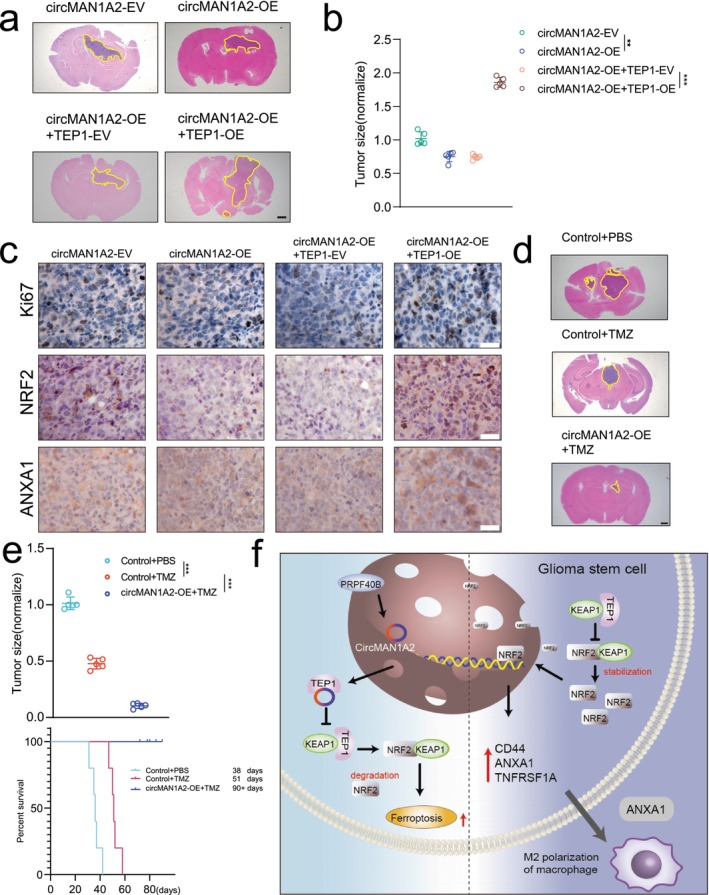
CircMAN1A2 suppresses glioma progression and promotes TMZ sensitive in vivo. (a) HE staining of brain gliomas in nude mice with circMAN1A2 knockdown or overexpression, and with circMAN1A2 overexpression followed by regulation of TEP1 expression. (b) Differences in intracranial tumor size of tumor‐forming tumors plotted for different treatment scenarios. (c) IHC staining plots for tumor detection of Ki67, NRF2, and ANXA1 under different treatments. (d) HE staining shows the growth of gliomas in the brain of hormonal mice in response to TMZ treatment under different treatment conditions. (e) Effect of TMZ treatment on tumor size and survival in hormonal mice under different treatment scenarios. (f) Schematic representation of the mechanism by which circMAN1A2 regulates GSC progression.

In the previous sections of our study, we confirmed through in vitro cell experiments that circMAN1A2 inhibits glioma stem cell proliferation and stemness while reversing TMZ resistance. Next, we further validated these findings and molecular expressions in in vivo experiments. We transfected the GL261 cell line with a lentivirus overexpressing circMAN1A2 and implanted it into C57 mice to observe the in vivo effects of circMAN1A2. Overexpression of circMAN1A2 inhibited glioma progression, an effect that could be blocked by the overexpression of TEP1, as evidenced by enlarged necrotic areas within the tumor observed through HE staining (Figure 7a,b). Subsequent immunohistochemistry (IHC) assays were performed to assess the expression levels of Ki67, NRF2, and ANXA1 in all groups. The results indicated that overexpression of circMAN1A2 significantly suppressed the expression of Ki67, NRF2, and ANXA1, while TEP1 overexpression restored their expression levels (Figure 7c). To assess the therapeutic effect of circMAN1A2 in combination with TMZ, we performed TMZ treatment experiments. The results demonstrated that circMAN1A2 overexpression significantly improved the efficacy of TMZ, prolonging the survival time of mice (Figure 7d,e). As depicted in Figure 7f, PRPF40B enhanced the expression of circMAN1A2, which in turn could bind to TEP1, preventing the binding of TEP1 to KEAP1. This competition led to the degradation of NRF2, promoting ferroptosis in GSCs and inhibiting tumor progression (left panel). In the absence of circMAN1A2, TEP1 bound to KEAP1, which released NRF2 and inhibited ferroptosis. Concurrently, NRF2 upregulated ANXA1, promoting the recruitment and M2 polarization of TAMs (right panel).

## 4 Discussion

Glioblastoma, the most malignant brain tumor, exhibits resistance to multiple therapies [7]. Although circRNAs are highly abundant in mammalian brain tissue, with highly conserved sequences and upregulated expression during neuronal differentiation [35], the role of downregulated circRNAs in gliomas remains poorly understood. Recent studies have highlighted the role of upregulated circRNAs in glioma tissues, promoting the malignant progression of glioblastoma stem cells [14, 36, 37]. In this study, by combining analysis of public datasets and clinical samples, we identified circMAN1A2 (located at chr1:117944807–117963271) as the most significantly downregulated circRNA in glioma tissues. Importantly, circMAN1A2 expression negatively correlated with WHO grade and emerged as a valuable prognostic marker for glioma patients. Functional studies using circMAN1A2‐manipulated GSC cell line models confirmed its tumor suppressive role in vitro, with overexpression inhibiting GSC proliferation and self‐renewal. Given the central role of GSCs in glioma recurrence and treatment resistance, we developed TMZ‐resistant GSC lines. Notably, circMAN1A2 expression was downregulated in these TMZ‐resistant GSCs. Furthermore, circMAN1A2 overexpression reversed their TMZ resistance and inhibited their proliferation and tumor cell stemness.

Ferroptosis is a programmed cell death distinct from apoptosis, primarily executed through iron‐mediated lipid peroxidation reactions [38]. This process is tightly regulated by several intracellular metabolic pathways, including those controlling cellular redox state, iron metabolism, and the metabolism of specific lipid compounds [39, 40, 41]. Multiple studies have shown that cancer cells resistant to traditional apoptosis‐based therapies, such as mesenchymal stem cells, dedifferentiated cancer cells, and highly treatment‐resistant “persistent cells”, display a high degree of sensitivity to ferroptosis [38]. Inhibition of SOAT1 regulates SLC40A1 through the PI3K/AKT/mTOR pathway, elevating the level of lipid peroxidation in glioma cells and enhancing their sensitivity to ferroptosis inducers and radiotherapy [42]. Additionally, the circRNF10/ZBTB48/IGF2BP3 positive feedback loop activation promotes the malignant progression of GSCs by inhibiting ferroptosis [43]. We employed a series of ferroptosis‐associated assays to determine that circMAN1A2 inhibits GSC malignant progression and reverses TMZ resistance by inducing GSC ferroptosis. Currently, circRNAs have been discovered to exert biological functions through various mechanisms, including acting as miRNA sponges to regulate downstream gene expression, binding proteins to modulate protein function, and even translating into polypeptides [44, 45, 46, 47]. As the field of circRNA research advances, researchers continue to uncover novel mechanisms of action for these molecules. For instance, circRNA CDR1as directly binds to the p53 DNA‐binding domain (DBD), preventing p53 ubiquitination and destabilizing the p53 protein by disrupting the formation of the p53/MDM2 complex. This ceRNA‐independent mechanism contributes to inhibiting the malignant progression of gliomas [44]. CircFoxo3 acts as a scaffold protein, promoting the formation of a complex between p21 and CDK2, thereby inhibiting CDK2 activity and impeding cell cycle progression [48]. In our study, we focused on identifying proteins that interact with circMAN1A2. By combining bioinformatics prediction with LC–MS/MS analysis, we identified TEP1, a protein with an unreported role in gliomas, as a downstream functional protein of circMAN1A2‐induced ferroptosis in GSCs. Interestingly, we found that circMAN1A2 does not regulate TEP1 mRNA and protein expression levels. Further experiments revealed that circMAN1A2 could directly bind to TEP1, preventing the binding of the TEP1‐KEAP1 and leading to NRF2 protein degradation.

The KEAP1‐NRF2 system plays a critical role in maintaining cellular homeostasis by acting as a major point of intracellular defense against both exogenous and endogenous oxidative stress [28]. Under normal conditions, the KEAP1‐CLU3 ubiquitin E3 ligase complex tags NRF2 for degradation by the proteasome. However, upon accumulation of reactive oxygen species in the cell, KEAP1 releases NRF2, leading to its cytoplasmic accumulation and subsequent nuclear translocation. In the nucleus, NRF2 activates an anti‐oxidative stress gene transcriptional program to restore cellular homeostasis [28, 49]. It is now understood that NRF2 is a key transcription factor not only for cellular adaptation and survival under oxidative stress but also for maintaining GSC stemness and self‐renewal. Accordingly, dysfunction of the KEAP1‐NRF2 pathway plays a significant role in the malignant progression of gliomas [50]. HACE1, for instance, promotes malignant progression and radiotherapy resistance in glioma by competitively binding to NRF2 with KEAP1, thereby enhancing NRF2 stability and IRES‐dependent upregulation of NRF2 translation levels, leading to a significant decrease in intracellular ROS levels [51]. Herein, we employed a combination of co‐immunoprecipitation with LC–MS/MS analysis and protein docking to identify TEP1 as a strong interactor with KEAP1. Interestingly, neither knockdown nor overexpression of TEP1 affected the mRNA or protein levels of KEAP1. In contrast, NRF2 protein levels and cellular localization changed significantly with TEP1 expression levels. These findings suggest that TEP1 regulates NRF2 protein levels primarily through competitive binding with KEAP1.

KEAP1 is a protein containing two key structural domains: the BTB/BRCT domain and the Kelch domain, which serves as the binding site for NRF2. The interaction between the Kelch domain and the Neh2 domain of NRF2 is facilitated by specific motifs within NRF2, including DLG and ET/SGE motifs [52]. Recent studies have identified several tumor‐associated proteins that competitively bind to KEAP1 via the ET/SGE motif, thereby regulating NRF2 activity. For instance, NESTIN relies on its own ESGE motif (amino acids 1412–1422) to competitively bind KEAP1 and promote NRF2 protein upregulation, contributing to malignant progression in non‐small cell lung cancer [53]. Similarly, DPP9 disrupts KEAP1‐NRF2 binding through its conserved ESGE motif, leading to NRF2‐dependent transcriptional activation of SLC7A11 and promoting tumorigenesis and drug resistance in renal clear cell carcinoma [54]. In our study, we observed that a stronger interaction between TEP1 and KEAP1 increased NRF2 protein stability. Given that DLG and ESGE motifs are critical for the binding of KEAP1‐NRF2, we analyzed the TEP1 protein sequence, which revealed the presence of two DLG motifs (residues 1837–1839 and 2369–2371) and one ESGE motif (residues 2443–2446). We focused on the ESGE motif due to its higher affinity for KEAP1 compared to the DLG motif [55]. Interestingly, deletion or mutation of the ESGE motif in TEP1 abolished its interaction with KEAP1, leading to NRF2 degradation. The presence of both DLG and ESGE motifs in TEP1 suggests a potential role in maintaining the stability of the intracellular environment in GSC cells. Further studies are warranted to fully elucidate the biological functions of TEP1 in GSCs.

We also identified PRPF40B, an upstream molecule previously unreported in the context of gliomas, as a regulator of circMAN1A2 expression. PRPF40B is a presumed mammalian ortholog of Prp40, generally involved in early spliceosome assembly. Current research suggests it may be associated with neurological diseases [56], but its study in tumors is still limited. Through clinical sample analysis and related experiments, we found that the expression of PRPF40B is negatively correlated with WHO grading and positively correlated with circMAN1A2. Therefore, it might serve as an upstream regulator of circMAN1A2. However, the specific mechanisms involved still require further in‐depth research in the future.

Tumor‐associated macrophages are the most abundant immune cells in GBM, accounting for 30%–50% of absolute tumor mass [57, 58]. Glioma cells can release a variety of chemokines to recruit TAMs and promote their transformation into the M2 phenotype. Interestingly, GSCs are able to recruit TAMs more efficiently than more differentiated glioma cells [59]. GSCs preferentially secrete POSTN proteins over non‐stem cell glioma cells. This secretion allows GSCs to recruit TAMs via integrin αvβ3 and maintain the immunosuppressive M2 phenotype of TAMs, thereby supporting GBM growth and angiogenesis [60]. Transcription factors act as master regulators of numerous genes, controlling cell types, developmental patterns, and specific cellular programs [32]. It has been reported that NRF2 in epidermal keratinocytes was found to drive macrophage movement toward wounds by upregulating the expression level of the secreted protein CCL2 in a repulsive manner [33]. We constructed the NRF2 gene regulatory network in glioma cells using single‐cell RNA sequencing data and identified ANXA1 as a downstream *NRF2* target gene. ANXA1 has been implicated in promoting treatment resistance and immunosuppressive microenvironment formation in glioma. Specifically, ANXA1 released from glioma cells can induce the M2 phenotype in macrophages by binding to the macrophage FPR1 receptor. Additionally, ANXA1 contributes to the generation of a Treg cell‐driven immunosuppressive microenvironment [61]. Hypoxia activation of the HIF1A‐FOSL2 axis in glioma cells upregulates ANXA1 recruitment and induces the transformation of monocytes into M2‐like macrophages. These M2‐like macrophages, in turn, significantly inhibit CD8+ T cell proliferation and IFNγ production, leading to the generation of an immunosuppressive microenvironment [62]. The mechanisms by which ANXA1 affects macrophage polarization in gliomas and other cancers have been well‐established in recent years [61]. Our experiments consistently demonstrated that GSC‐derived ANXA1 promoted THP1‐induced macrophage migration and M2 phenotypic polarization, which is consistent with the findings of Zheng et al. Overexpression of ANXA1 or the addition of recombinant ANXA1 protein significantly restored the decreased macrophage migration capacity and decreased M2 phenotypic markers caused by NRF2 knockdown. These findings suggest that ANXA1 functions as a bridge for NRF2 participation in GSC‐TAM interactions, highlighting the importance of ANXA1 for tumor‐associated macrophage infiltration and M2 phenotype polarization. It is important to note that innate immunity plays a crucial role in tumor progression, and existing studies have shown that circRNAs can regulate the activation of innate immune proteins and the activation of immune cells. Our in vivo experimental model uses BALB/c nude mice. While circRNAs may influence tumor progression through other, more complex mechanisms by regulating innate immunity, in this study, we primarily focus on the regulatory processes within tumor cells. The mechanism by which circMAN1A2 functions in innate immunity is an area we plan to explore further in future research.

Finally, we verified the impact of circMAN1A2 on tumor progression and TMZ treatment in vivo, consistent with the results of the in vitro assays. Overexpression of circMAN1A2 inhibited tumor progression and increased tumor efficacy to TMZ treatment. These results suggest the potential value of circMAN1A2 as a target for GBM therapy in clinical applications. It should be noted that most of the data on TMZ resistance in this study were obtained from in vitro experiments. It is well known that in vitro and in vivo pharmacokinetics differ. Although we validated the effect of circMAN1A2 on TMZ treatment in vivo, we did not further investigate the more complex aspects of in vivo drug metabolism, which is a limitation of this study. In future research, we will focus more on the mechanisms of in vivo drug metabolism and conduct more in‐depth studies on the impact of circMAN1A2 on TMZ treatment. Meanwhile, we observed changes in NRF2 and ANXA1 expression levels that correlated with circMAN1A2 expression. It is now understood that circRNAs, due to their greater stability compared to traditional RNA, can function continuously. Our findings provide a novel molecular target for targeting GSCs by inducing ferroptosis, offering a promising strategy for GBM treatment.

## 5 Conclusion

In conclusion, our study unveils circMAN1A2 as a novel suppressor of the malignant GSC phenotype by inducing ferroptosis. We comprehensively elucidate the specific molecular mechanism underlying this process, demonstrating how circMAN1A2 directly interacts with TEP1 to promote ferroptosis. Furthermore, we reveal a hitherto undocumented function of TEP1 in GSCs, distinct from its known roles in telomerase activity regulation and RNA binding. Additionally, we identify *ANXA1* as a novel downstream target gene of *NRF2* and demonstrate its role as an intermediary molecule mediating NRF2 involvement in GSC‐TAM interaction. Given the potential of circRNAs as targets for nanoplatform drug delivery systems and the ongoing advancements in artificial circRNA synthesis technology, our findings offer a promising new avenue for GBM treatment.

## Author Contributions

Z.J. conceived and designed the study; X.L., J.H., and W.Z. performed the experiments and collected the data; Z.F., Y.W., and H.C. performed bioinformatics analysis and analyzed the data. H.L. and X.L. interpreted results and wrote the manuscript. All authors read and approved the final version of the manuscript.

## Ethics Statement

The experimental protocol was reviewed and approved by the Laboratory Animal Ethics Committee of China Medical University (KT2022436), and the experiments were performed in the SPF laboratory. Patients and controls were acquired with informed consent, under the protocol approved by the First Hospital of China Medical University research ethics committee (AF‐SOP‐07‐1.2‐01).

## Consent

Consent to publish has been obtained from all authors.

## Conflicts of Interest

The authors declare no conflicts of interest.

## Supporting information


Figure S1

Table S1

Table S2

Table S3

Table S4

Table S5

## Data Availability

The datasets obtained and analyzed during the current study were made available from the corresponding authors through request. The data are not publicly available due to privacy or ethical restrictions.
