# Supplementary material for: CircMAN1A2 Levels Determine GBM Susceptibility to TMZ in a Pathway Involving TEP1‐ and KEAP1‐Mediated NRF2 Degradation Leading to Ferroptosis
Source: CNS Neurosci Ther. 2025 Jun 30;31(7):e70489. doi: 10.1111/cns.70489 (PMC12207318; doi:10.1111/cns.70489)
Supplement: Supplementary file 1 — Figure S1 [file CNS-31-e70489-s003.pdf]

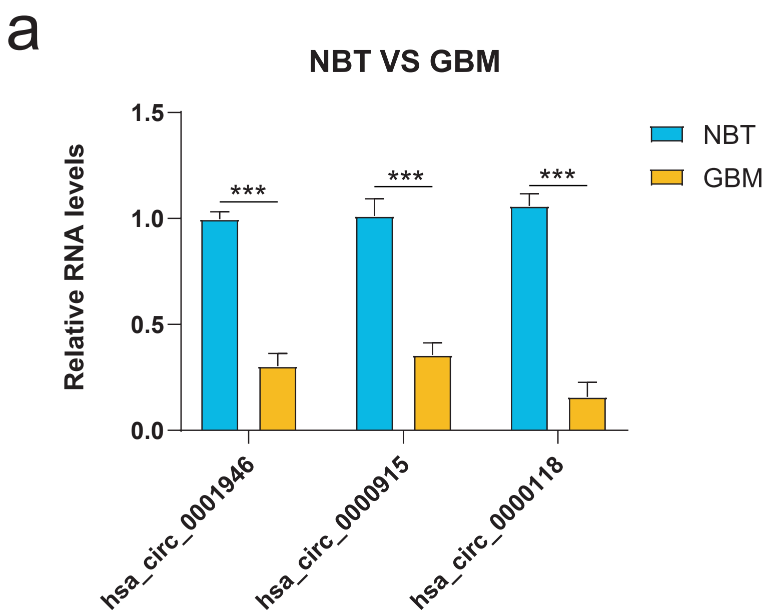

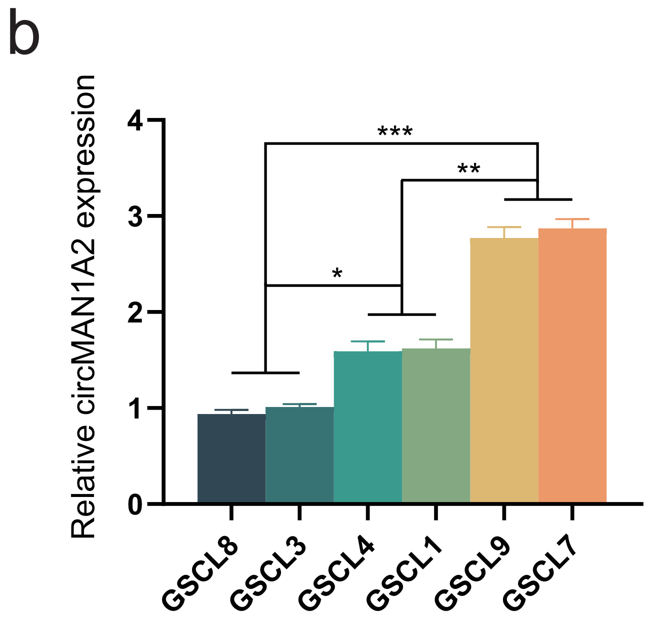

Supplementary figure 1.
a.Compared the expression levels of circRNAs in normal tissues and GBM, and the results showed that hsa_circ_0000118 (circMAN1A2) was down-regulated at a much lower level in GBM.
b.Comparison of RNA expression of circMAN1A2 in GSCL1, GSCL3, GSCL4, GSCL7, GSCL8 and GSCL9.
All results are expressed as SD ± mean (three independent experiments). $*p < 0.05$; $**p < 0.01$; $***p < 0.001$; $****p < 0.0001$.

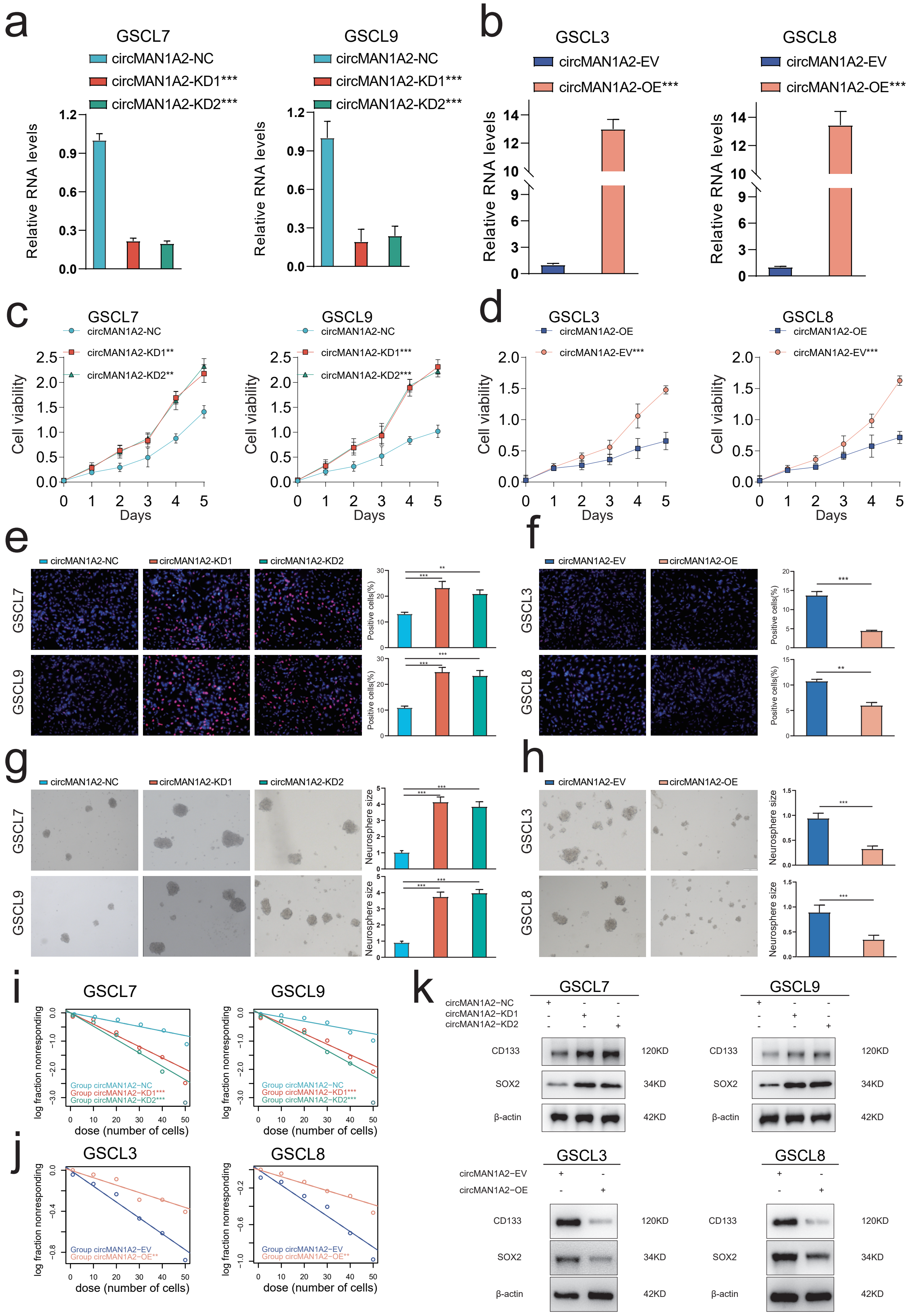

Supplementary figure 2.

a,b. qPCR verified the effects of knockdown (a) and overexpression (b) of circMAN1A2.

c,d. MTS assay reveals GSC cell viability after knockdown (c) and overexpression (d) of circMAN1A2.

e,f. Edu assay showing proliferation of different GSC motifs after circMAN1A2 knockdown (e) or overexpression (f) treatment. Scale bar = 100 um.

g,h. Neurosphere formation assay reveals the size of neurosphere in different GSC groups after circMAN knockdown (g) or overexpression (h) treatment. Scale bar = 20 um.

i,j. The limiting dilution assays revealed the neurosphere formation capacity of different GSC groups after circMAN1A2 knockdown (i) or overexpression (j) treatment.

k. Western blot reveals invasive changes in GSC cells after knockdown and overexpression of circMAN1A2.

All results are expressed as SD ± mean (three independent experiments). *$p < 0.05$; **$p < 0.01$; ***$p < 0.001$; ****$p < 0.0001$.

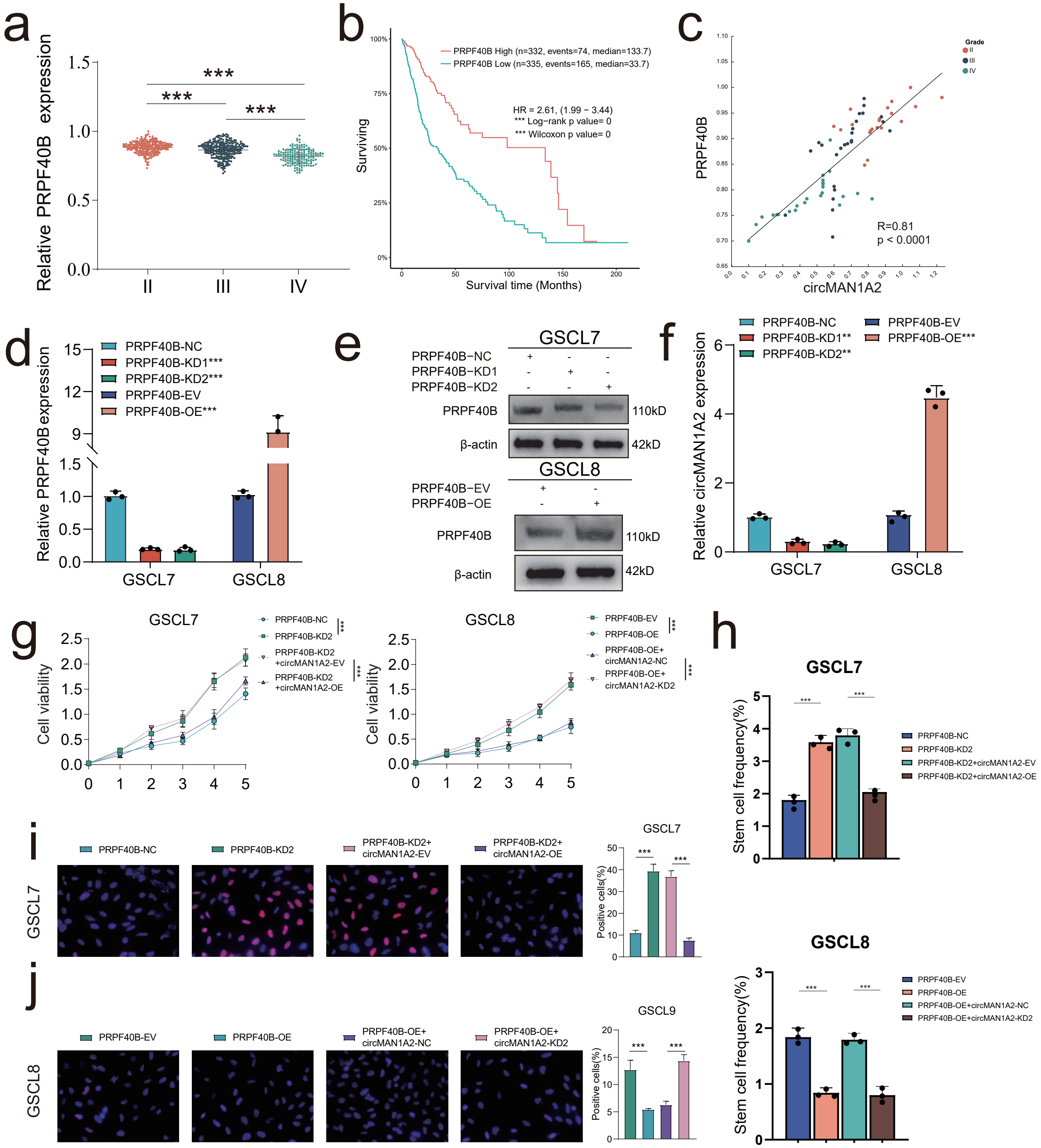

Supplementary figure 3.

a.Expression of PRPF40B in gliomas of different grades, indicating its varying presence in tumor severity.

b.Kaplan-Meier prognostic survival curves for PRPF40B in glioma patients, suggests that PRPF40B expression and prognosis are positively correlated (***p<0.001).

c.Correlation of PRPF40B and circMAN1A2 expression, suggesting a regulatory relationship.

d.qRT-PCR validation of knockdown of PRPF40B in GSCL7 cell line (left) and overexpression of PRPF40B in GSCL8 cell line (right), Indicates successful knockout or overexpression (***p<0.001).

e.Western blot validation of knockdown of PRPF40B in GSCL7 cell line (upper) and overexpression of PRPF40B in GSCL8 cell line (lower).

f.qRT-PCR was performed to detect changes in circMAN1A2 after knockdown of PRPF40B in GSCL7 cell line (left) and overexpression of PRPF40B in GSCL8 cell line (right), suggests that circMAN1A2 expression is positively correlated with PRPF40B expression.

g-h. MTS (g), limiting dilution assays (h) and EDU (i and j) were performed to detect malignant phenotypic changes in cells after knockdown of PRPF40B in the GSCL7 cell line and overexpression of PRPF40B in the GSCL8 cell line.

All results are expressed as SD ± mean (three independent experiments). *p < 0.05; **p <0.01;***p < 0.001; ****p < 0.0001.

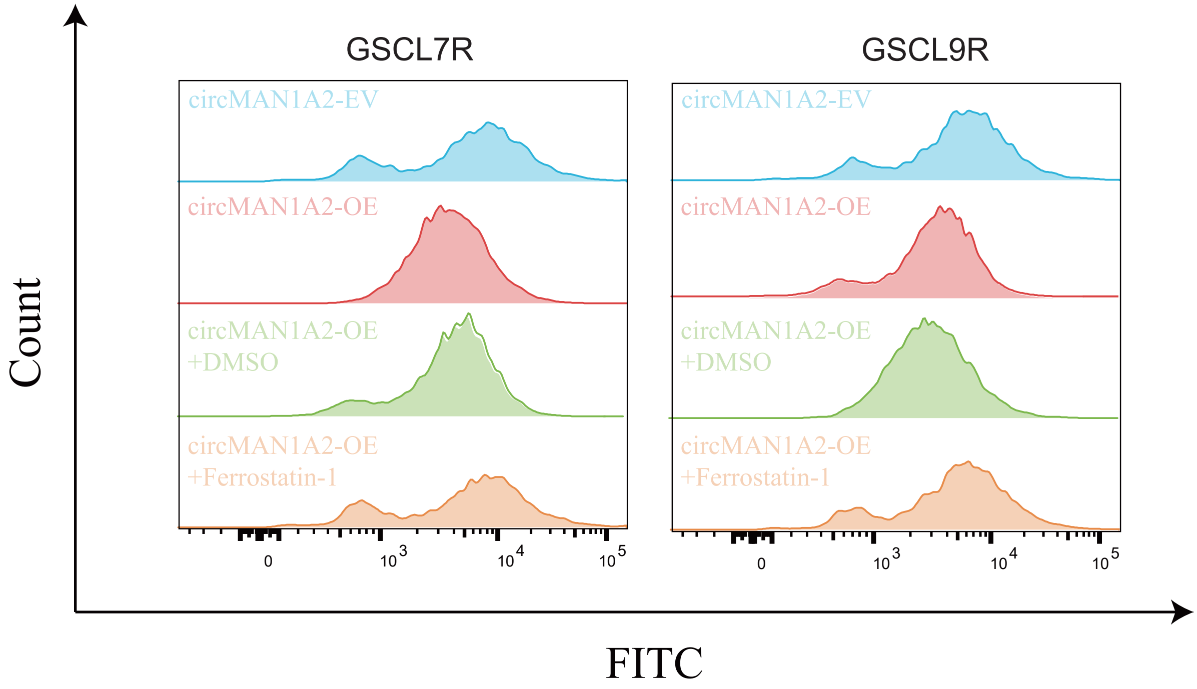

Supplementary figure 4.

Detection of ROS levels in GSCL7R and GSCL9R cells after the addition of Fer-1.

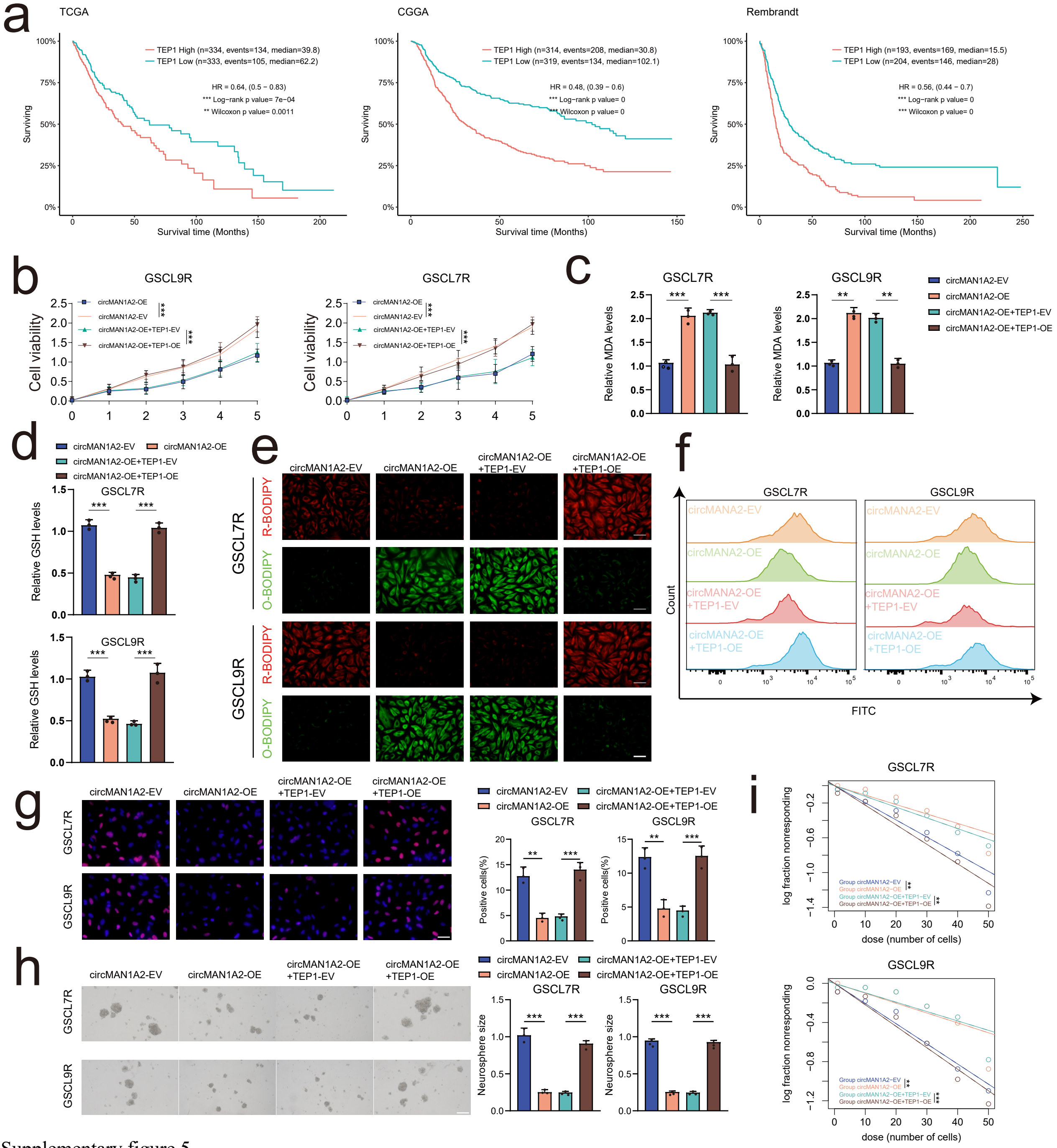

Supplementary figure 5.

a.Survival curves of TEP1 in TCGA cohort, CGGA cohort and Rembrandt cohort.

b.MTS assay for cell viability after overexpression of TEP1 in circMAN1A2 overexpression-treated TMZ-resistant cell lines (GSCL9R, GSCL7R).

c.MDA levels were detected after overexpression of TEP1 in circMAN1A2 overexpression-treated TMZ-resistant cell lines (GSCL9R, GSCL7R).

d.GSH levels were detected after overexpression of TEP1 in circMAN1A2 overexpression-treated TMZ-resistant cell lines (GSCL9R, GSCL7R).

e.BODIPY detects changes in lipid peroxidation levels after overexpression of TEP1 in circMAN1A2 overexpression-treated TMZ-resistant cell lines (GSCL9R, GSCL7R).

f. Detection of ROS levels in circMAN1A2 overexpression-treated TMZ-resistant cell lines (GSCL9R, GSCL7R).

g-i. EDU, neurosphere formation assay and limiting dilution assa y were performed to detect malignant phenotypic changes in circMAN1A2 overexpression-treated TMZ-resistant cell lines (GSCL9R, GSCL7R).

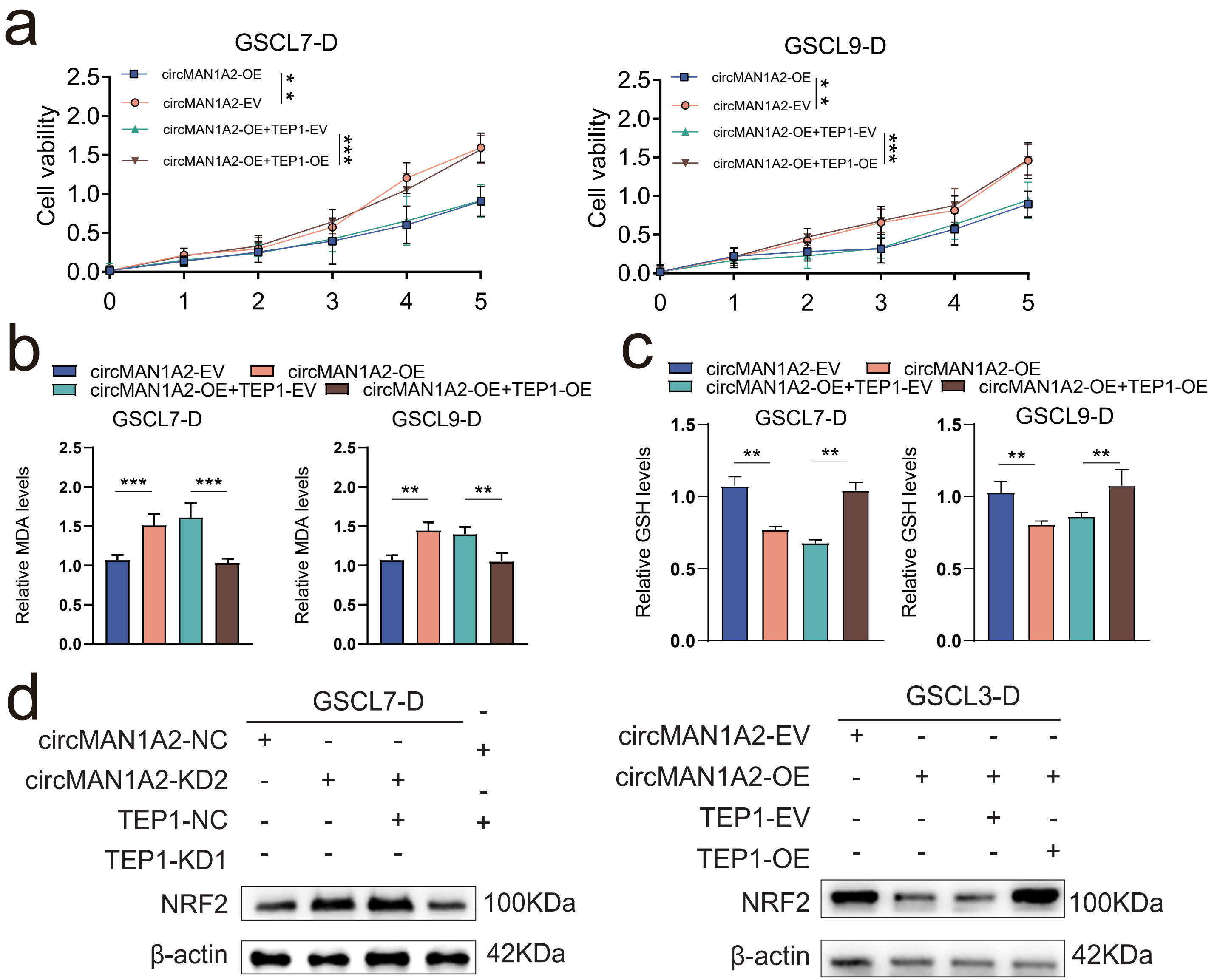

Supplementary Figure 6.

a.MTS assay for cell viability after overexpression of TEP1 in circMAN1A2 overexpression-treated TMZ-resistant cell lines (GSCL9-D, GSCL7-D).

b.MDA levels were detected after overexpression of TEP1 in circMAN1A2 overexpression-treated TMZ-resistant cell lines (GSCL9-D, GSCL7-D).

c.GSH levels were detected after overexpression of TEP1 in circMAN1A2 overexpression-treated TMZ-resistant cell lines (GSCL9-D, GSCL7-D).

d.Western blotting was performed to detect changes in the expression level of NRF2 protein in circMAN1A2 knockdown TEP1 overexpression and circMAN12 overexpression TEP1 knockdown GSC defferentiated cell lines, respectively.

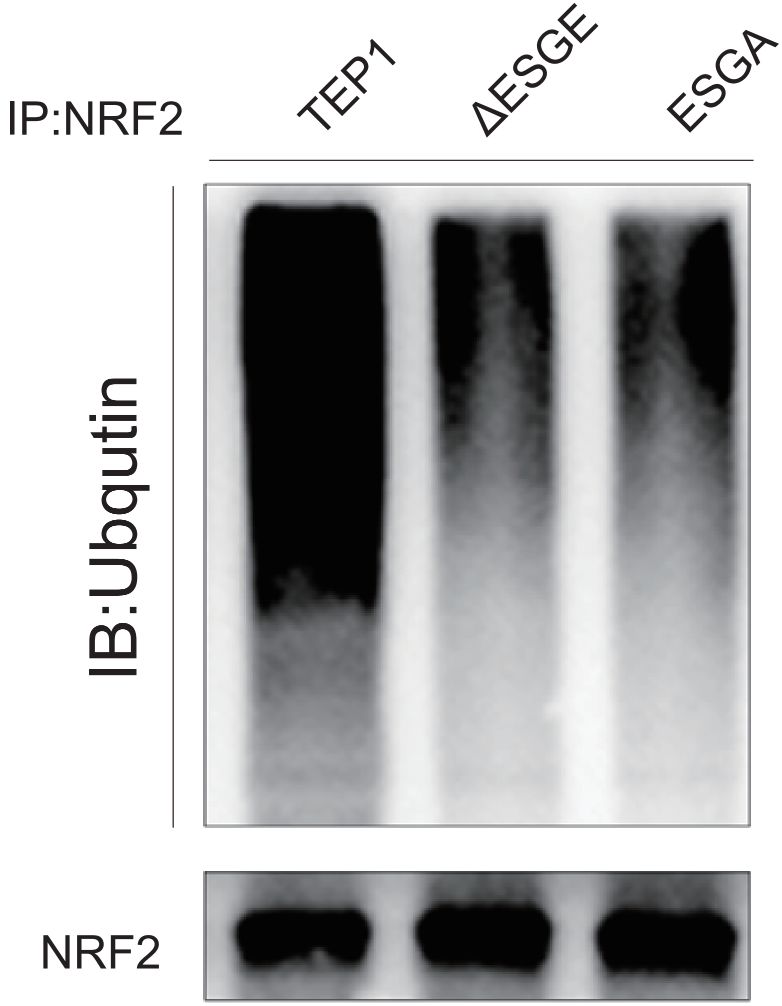

Supplementary figure 7.
In vitro ubiquitination results indicate that the deletion or mutation of the ESGE motif in TEP1 reduces the ubiquitination level of NRF2.

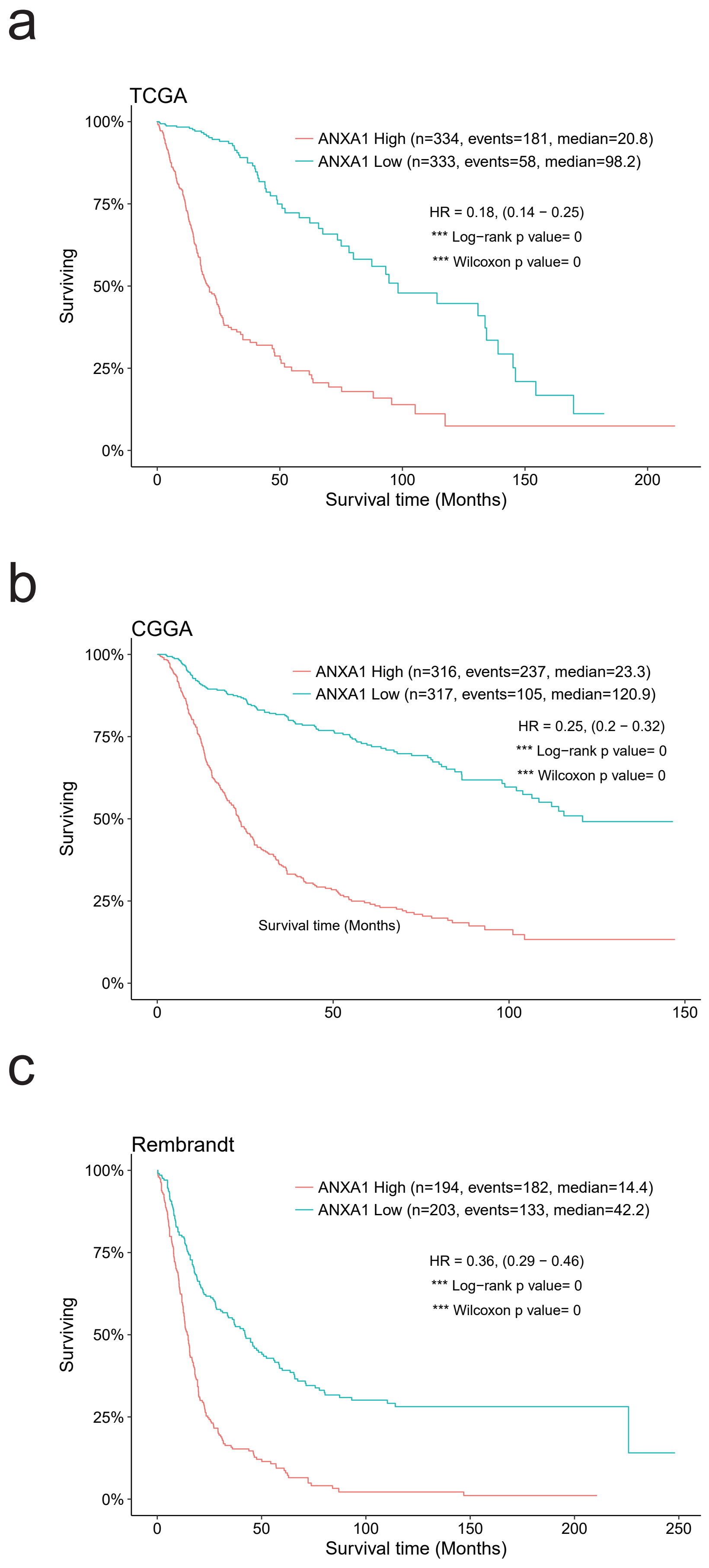

Supplementary figure 8.
a.Survival curves of ANAX1 in TCGA cohort.
b.Survival curves of ANAX1 in CGGA.
Survival curves of ANAX1 in Rembrandt cohort.

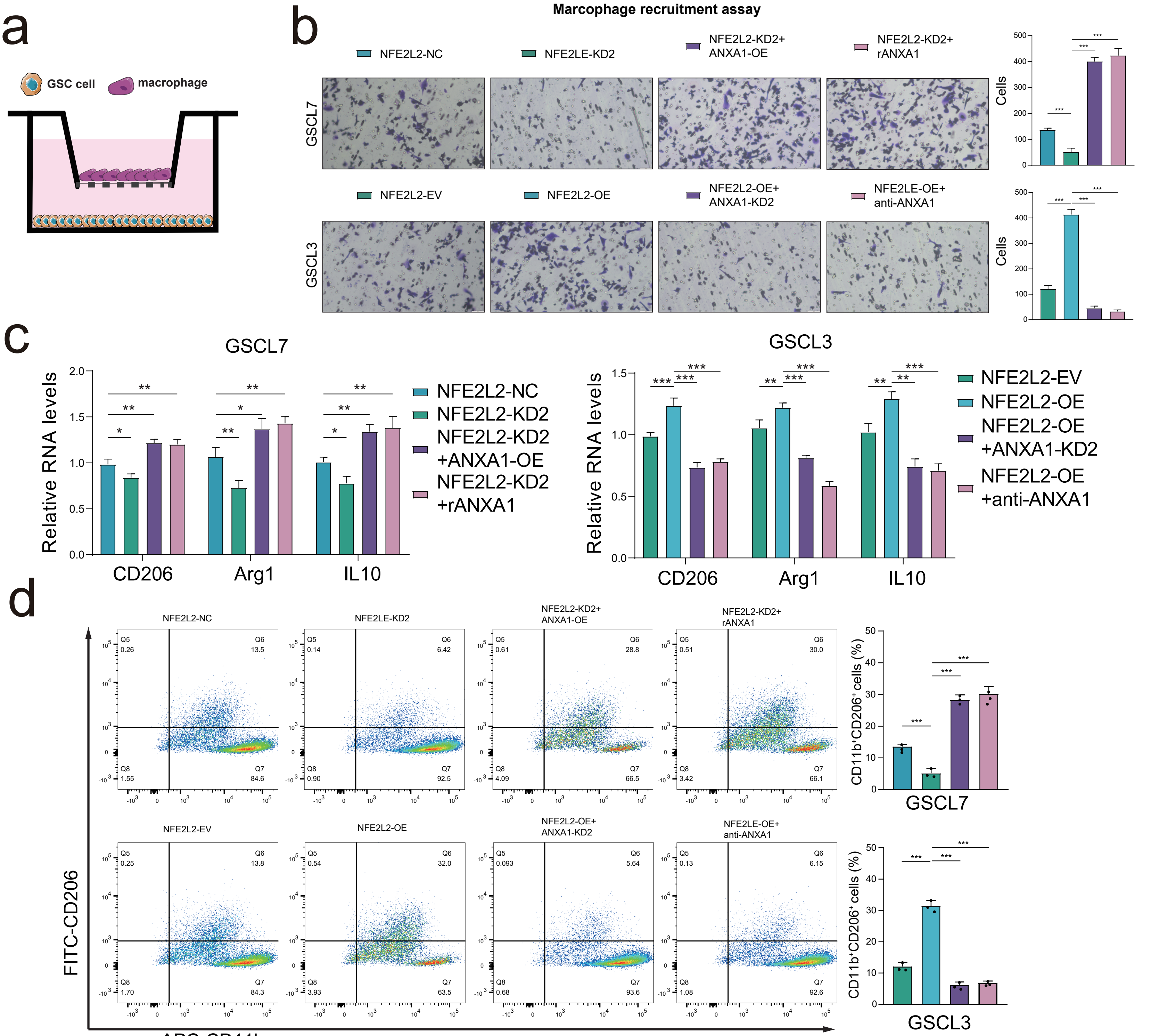

Supplementary Figure 9.

a. Co-culture model diagram.

b. Transwell assay shows TAM infiltration levels.

c. The qRT-PCR assay showed the expression levels of the M2 phenotypic markers CD206, Arg1, and IL10 genes of TAM.

d. Flow cytometry was used to detect the proportion of macrophage polarization.
