# Supplementary material for: CircMAN1A2 Levels Determine GBM Susceptibility to TMZ in a Pathway Involving TEP1‐ and KEAP1‐Mediated NRF2 Degradation Leading to Ferroptosis
Source: CNS Neurosci Ther. 2025 Jun 30;31(7):e70489. doi: 10.1111/cns.70489 (PMC12207318; doi:10.1111/cns.70489)
Supplement: Supplementary file 2 — Table S1 [file CNS-31-e70489-s005.pdf]

Table S1 Relationship of circMAN1A2 expression to clinical features of glioma patients

Clinical features		Samples (n=70)	CircMAN1A2 expression*		P-value
			**Low(n=35)**	**High(n=35)**	
Sex	Male	46	22	24	P=0.912
	Female	24	13	11	
WHO grade	Ⅱ	20	2	18	P＜0.001
	Ⅲ	25	21	4	
	Ⅳ	25	22	3	
IDH status	Wild	30	21	9	P=0.017
	Mutant	40	14	26	
1p/19q status	Codeletion	25	8	17	P=0.075
	Non-codeletion	45	17	18	

*CircMAN1A2 expression was detected by RT-qPCR and ranked from low to high. The high expression of circMAN1A2 defined as the expression level higher than the median expression level of circMAN1A2.
