# Supplementary material for: CircMAN1A2 Levels Determine GBM Susceptibility to TMZ in a Pathway Involving TEP1‐ and KEAP1‐Mediated NRF2 Degradation Leading to Ferroptosis
Source: CNS Neurosci Ther. 2025 Jun 30;31(7):e70489. doi: 10.1111/cns.70489 (PMC12207318; doi:10.1111/cns.70489)
Supplement: Supplementary file 5 — Table S4 [file CNS-31-e70489-s002.pdf]

qPCR Primers

Primer	Forward (5'-3')	Reverse (5'-3')
circMAN1A2	TGTGAATTCAGAGGTGTCTGTG	GCTTCTTCCAAGGCCTTCTC
PRPF40B	CCAATGCTTCCACCAATGGG	CACGGACTGCTTGTCGTCA
TEP1	CCACCCTCTCTAGTCTAAAGAGC	CAGCTTGCGTCATGTGAGATA
KEAP1	CTGGAGGATCATACCAAGCAGG	GGATACCCTCAATGGACACCAC
NRF2/NFE2L2	TCAGCGACGGAAAGAGTATGA	CCACTGGTTTCTGACTGGATGT
ANXA1	GCGGTGAGCCCCTATCCTA	TGATGGTTGCTTCATCCACAC
GAPDH	GGAGCGAGATCCCTCCAAAAT	GGCTGTTGTCATACTTCTCATGG
β-actin	CATGTACGTTGCTATCCAGGC	CTCCTTAATGTCACGCACGAT

siRNA sequences

Primer	Forward (5'-3')	Reverse (5'-3')
circMAN1A2-KD1	UGAAAUCAAGGUUGUCUUCAA	GAAGACAACCUUGAUUUCAGU
circMAN1A2-KD2	UCGAAUGUUGACUUCAAACAC	GUUUGAAGUCAACAUUCGAUU
PRPF40B -KD1	UUUACUCUGGUUGUUAUAGUA	CUAUAACAACCAGAGUAAAGA
PRPF40B -KD2	UUUUCUCACUCAGUUUAGGCA	CCUAAACUGAGUGAGAAAAAG
TEP1-KD1	UAUUGUUCAUGUUUACUUCUG	GAAGUAAACAUGAACAAUACA
TEP1-KD2	UUUUCCAUGGCUGAAACUCAG	GAGUUUCAGCCAUGGAAAAAC
NFE2L2-KD1	UGAAUGUUUGUCUUUUGUGAA	CACAAAAGACAAACAUUCAAG
NFE2L2-KD2	UCAUUUUCAAUAUUAAGACAC	GUCUUAAUAUUGAAAAUGACA
ANXA1-KD1	UUUAUCUCUACCUUCUUGCAA	GCAAGAAGGUAGAGAUAAAGA
ANXA1-KD2	UUUCUUCAGUGUUUCAUCCAG	GGAUGAAACACUGAAGAAAGC
