# Supplementary material for: CircMAN1A2 Levels Determine GBM Susceptibility to TMZ in a Pathway Involving TEP1‐ and KEAP1‐Mediated NRF2 Degradation Leading to Ferroptosis
Source: CNS Neurosci Ther. 2025 Jun 30;31(7):e70489. doi: 10.1111/cns.70489 (PMC12207318; doi:10.1111/cns.70489)
Supplement: Supplementary file 6 — Table S5 [file CNS-31-e70489-s006.pdf]

Table S5 The antibody information used in this research

Antibodies	Sourse
CD133	ProteinTech
TEP1	ProteinTech
NRAF2	ProteinTech
KEAP1	ProteinTech
SOX2	ProteinTech
Lamin B1	Abcam
PRPF40B	GeneTex
Ubiquitin	ProteinTech
β-actin	ProteinTech
IgG	Abcam
